# Benchmark of chromatin–protein interaction methods in haploid round spermatids

**DOI:** 10.3389/fcell.2025.1572405

**Published:** 2025-05-13

**Authors:** Ruolei Wang, Yue Wu, Ze Zhou, Yicheng Ma, Weidong Zhang, Zihang Wang, Weihan Luo, Peng Hua

**Affiliations:** State Key Laboratory of Reproductive Medicine and Offspring Health, Nanjing Medical University, Nanjing, China

**Keywords:** ChIP-seq, CUT&Tag, CUT&RUN, signal-to-noise, peaks, transcription factors, histone modification

## Abstract

**Introduction:**

Chromatin–protein interactions are fundamental for regulation of gene transcription. While chromatin immunoprecipitation followed by deep sequencing (ChIP-seq) has long been the gold standard for mapping these interactions, emerging techniques such as CUT&RUN and CUT&Tag, which offer advantages such as low-input requirements and high signal-to-noise ratios, have aroused great attention. However, research addressing the potential biases introduced by enzyme-based tagmentation approaches and comparative assessment with ChIP-seq remain absent.

**Methods:**

This study aims to systematically evaluate and compare the performance of ChIP-seq, CUT&Tag, and CUT&RUN for profiling genome-wide transcription factors and histone modification binding.

**Results:**

Our analysis revealed that all three methods reliably detect histone modifications and transcription factor enrichment, with CUT&Tag standing out for its comparatively higher signal-to-noise ratio. Detailed peak comparison revealed unique and overlapping enrichment among the three techniques. Additionally, CUT&Tag can identify novel CTCF peaks compared with the other two methods. A strong correlation was observed between CUT&Tag signal intensity and chromatin accessibility, highlighting its ability to generate high-resolution signals in accessible regions.

**Discussion:**

The systematic comparison summarizes the differences between CUT&Tag and CUT&RUN in terms of the signal-to-noise ratio and bias toward accessible chromatin. Considering the experimental procedures, signal specificity, and inherent biases, we recommend tailoring the choice of method to the type of chromatin–protein interaction under study. CUT&Tag offers a promising alternative for applications requiring high sensitivity and reduced background noise.

## Introduction

An understanding of the regulatory mechanisms governing gene expression propelled development in technologies that detect functional proteins that bind to genomic DNA. Over the past 2 decades, chromatin immunoprecipitation followed by deep sequencing (ChIP-seq) has become the most widely used method for profiling chromatin–protein interactions, offering comprehensive, genome-wide maps of transcription factor occupancy and histone post-translational modifications across various tissues and cell types. Simultaneously, the emergence of footprint research puts forward the need for precise detection of TFs and modified histone positioning, driving numerous technical innovations (Landt et al., 2012; Park, 2009).

**FIGURE 1:**
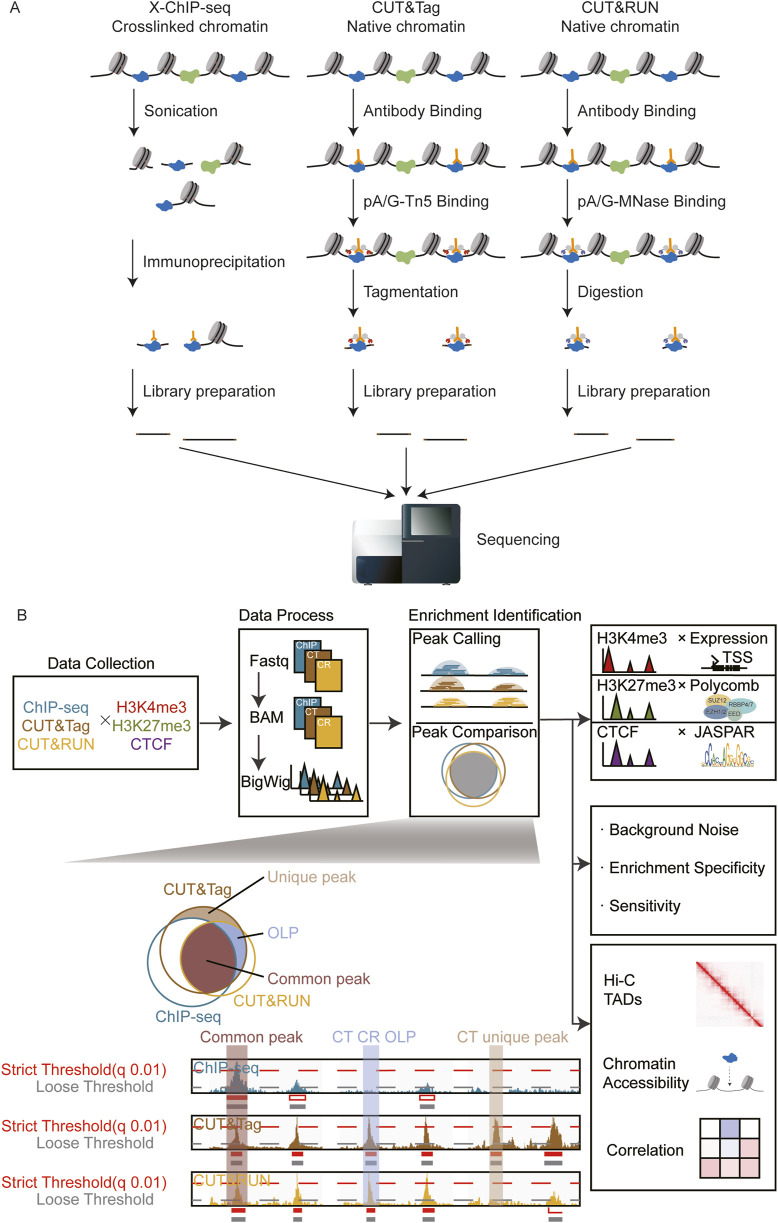
Overview of the research. **(A)** Library construction principles of ChIP-seq, CUT&Tag, and CUT&RUN. **(B)** Schematic diagram: systematic comparison of peak identification performance across three omics techniques.

Conventional ChIP-seq utilized formaldehyde to crosslink proteins and chromatin. Subsequent steps such as sonication and antibody pull-down are often accompanied by material loss and false-positive signals, thereby reducing the signal-to-noise ratio and raising concerns about the authenticity (Teytelman et al., 2013; Baranello et al., 2016; Meyer and Liu, 2014). Recent advancements in genome-wide methods for detecting chromatin-associated proteins on DNA primarily rely on *in situ* immune-cleavage, followed by deep sequencing. Cleavage under targets and release using nuclease (CUT&RUN) and cleavage under targets and tagmentation (CUT&Tag) exemplify these strategies (Skene and Henikoff, 2017; Kaya-Okur et al., 2019). These approaches employ enzymatic reactions to isolate short chromatin fragments surrounding the target protein. Specific enrichment effectively eliminates the interference of non-specific DNA fragments, thereby minimizing background noise. CUT&RUN utilized endonuclease and exonuclease activities of pA/G-MNase to cleave double-strand DNA around the target protein. CUT&Tag is similar but uses the pA-Tn5 enzyme rather than pA/G-MNase, thereby simplifying the experimental procedures (Kaya-Okur et al., 2019). As depicted in the schematic diagram, all three methods exhibit distinctive characteristics (Figure 1). Accordingly, various approaches of data analysis for CUT&RUN and CUT&Tag were reported (Yashar et al., 2022; Henikoff et al., 2023; Liu et al., 2023a), along with the algorithm benchmark (Cheng et al., 2024), expanding the diverse applications in different research contexts.

Although crosslink-independent and enzyme-specific fragmentation used in CUT&RUN and CUT&Tag offer well-documented advantages over ChIP-seq, including reduced cell inputs, improved signal-to-noise ratios, and lower sequencing depth requirement (Skene and Henikoff, 2017; Kaya-Okur et al., 2019), critical gaps remain in the understanding of their comparative performance. Verifying each detected signal through molecular biology experiments is impractical, especially when the scope of analysis is expanded to genome-wide contexts. Second, despite numerous pairwise comparisons in the literature, systematic evaluations of all three methods under standardized conditions are lacking, particularly regarding their library preparation efficiencies, analytical pipelines, and method-specific artifacts.

To address this, we conducted an in-depth analysis focusing on the well-characterized histone modifications H3K27me3 and H3K4me3, along with a transcription factor CTCF—a zinc finger protein involved in diverse cellular processes. This analysis provided a theoretical framework for selecting appropriate experimental protocols. Given the potential biases in sequencing read counts introduced by allele-specific binding (ASB), which influenced transcription factor binding efficiency and histone modification enrichment in polymorphic regions (Abramov et al., 2021; Hartwig et al., 2023; Matsuwaka et al., 2025), we selected round spermatids as our research model.

Additionally, we collected published ChIP-seq data using round spermatids to conduct a comparative analysis of the three methods. The results highlighted the advantages of CUT&Tag in detecting transcription factors as it provided high-resolution maps of histone modifications and CTCF binding in haploid cells. This study enhanced the understanding of protein–DNA binding detection techniques from a data-driven perspective, guiding researchers in selecting the most appropriate library construction method for different cell types, proteins, or transcription factors.

## Materials and methods

### Biological material preparation and immunofluorescence staining

Round spermatids were separated from testis of adult C57B6J mice (8 weeks), using counterflow centrifugal elutriation (CCE) methods (Barchi et al., 2009). A total of 104 round spermatids were collected and fixed with 4% paraformaldehyde (PFA) for 15 min and then inoculated on Polysine^TM^ microscopic slides. After a 5-min wash with Tris-Buffered Saline with Tween 20 (TBST), the cells were blocked with 5% bovine serum albumin (BSA) at room temperature for 1 h. Subsequently, the cells were incubated with peptide nucleic acid (PNA) (Vector Labs, RL-1072) for 1 h at room temperature and washed three times with TBST. Antifade reagent containing 4',6-diamidino-2-phenylindole (DAPI) (Invitrogen, P36935) was applied, and the samples were sealed with a coverslip. Imaging was performed using a Zeiss LSM 800 Confocal Laser Scanning Microscope. The stage of the cells was identified based on acrosome angles and nucleus shapes. The purity of round spermatids reached 95%. The following antibodies were used: H3K27me3 (Cell Signaling Technology, 9733s), H3K4me3 (Merck, 07-473), and CTCF (Abcam, ab70303).

### CUT&Tag library generation and sequencing

CUT&Tag experiments were performed with the Hyperactive Universal CUT&Tag Assay Kit for Illumina Pro (Vazyme Biotech, TD904). Briefly, cells were co-incubated with 5 μL pre-activated ConA beads in 1.5-mL low-binding tubes. Antibody buffer with 0.5∼1 μg antibody was added and incubated with cells at 4°C overnight. Cells were washed in dig-wash buffer and incubated with secondary antibodies for 1 h. After washing with 600 μL dig-wash buffer, 100 μL Dig-300 buffer containing 04 μM pA/G-Tnp Pro was added to samples and then rotated end-over-end at 25°C for 1 h. The antibody–target fragmented DNA was then released and purified with DNA clean beads. To construct the DNA library for sequencing, the amplification system in the PCR tube format was set: DNA products were bound to 7.5 μL DNA extract beads Pro. Then, 12.5 μL 2 × CAM mix and 5 μL N5N7 primer were added to a total reaction volume of 25 μL. The samples were amplified with the following PCR program: initial extension at 72°C for 3 min, followed by an initial denaturation at 95°C for 3 min. The reaction proceeded through 14 cycles of denaturation at 98°C for 10 s, annealing at 60°C for 5 s, and extension at 72°C for 1 min. PCR products were purified with 1.8 × volume clean beads and eluted with ddH_2_O. The final size distributions were assessed by Agilent 2100 TapeStation for quality control before sequencing. Paired-end Illumina sequencing was conducted using the NovaSeq 6000 PE150 strategy.

### CUT&RUN library generation and sequencing

CUT&RUN experiments were performed with a Hyperactive pG-MNase CUT&RUN Assay Kit for Illumina (Vazyme Biotech, HD102). Cells were incubated with pre-washed ConA Beads Pro. Antibody buffer with 0.5 μg antibody was added and cultured with cells at 4°C overnight. Cells were treated and fragmented according to the kit protocol. DNA products underwent damage repair and end preparation, and the kit-provided adapters were added to the end. The libraries were amplified with 10 μL purified adapter ligation products, 2.5 μL PCR primer mix, and 12.5 μL HiFi amplification mix. Samples were amplified with the following PCR program: initial extension at 72°C for 3 min, followed by an initial denaturation at 95°C for 3 min. The reaction proceeded through 14 cycles of denaturation at 98°C for 10 s, annealing at 60°C for 5 s, and extension at 72°C for 1 min. PCR products were purified with 1.8 × volume clean beads and eluted with ddH_2_O. The final size distributions were assessed by using Agilent 2100 TapeStation for quality control before sequencing. Paired-end Illumina sequencing was conducted using the NovaSeq 6000 PE150 strategy.

### ATAC-seq library generation and sequencing

ATAC-seq experiments were performed with a TruePrep DNA Library Prep Kit V2 for Illumina (Vazyme Biotech, TD501). Cells were first incubated and permeabilized with 5% Triton X-100 and 0.5% digitonin. Genome DNA was fragmented using TruePrep Tagment Enzyme (TTE). The DNA products were purified with a FastPure Gel DNA Extraction Mini Kit (Vazyme Biotech, DC301) and amplified with the following PCR program: initial extension at 72°C for 3 min, followed by an initial denaturation at 98°C for 30 s. The reaction proceeded through 14 cycles of denaturation at 98°C for 15 s, annealing at 60°C for 30 s, and extension at 72°C for 3 s.

### Data collection

Omics data of round spermatids and diploid cell lines were downloaded from the Gene Expression Omnibus (GEO) database. Mouse round spermatids; RNA-seq; ChIP-seq for H3K4me3 and H3K27me3: GSE42629 and GSE214316 (Erkek et al., 2013; Gill et al., 2023); Hi-C: GSE147536 (Guo et al., 2020). Human embryonic kidney 293 cells (HEK293T), ChIP-seq, CUT&Tag, CUT&RUN for H3K4me3: GSE213209, GSE223370, GSE183730 (Chen et al., 2023; Esain-Garcia et al., 2024; Hogan et al., 2021) and ATAC-seq: GSE270033 (Niu et al., 2023). Mouse embryonic stem cell (mESC) E14Tg2a, ChIP-seq, CUT&Tag, and CUT&RUN for H3K27me3: GSE206735, GSE253062, GSE193910 (Ibarra et al., 2022), and ATAC-seq: GSE231410 (Miyazaki et al., 2023). Myelogenous leukemia cell line K562, ChIP-seq, CUT&Tag, CUT&RUN for CTCF: GSE92881, GSE124557, GSE151326 (Kaya-Okur et al., 2019; Schuijers et al., 2018; Lai et al., 2021), and ATAC-seq: GSE250133 (Corces et al., 2017). Supplementary Table S3 details all antibodies used in the GEO datasets.

### RNA-seq data analysis

Raw sequencing reads were first trimmed with fastp (0.20.0) (Chen et al., 2018) to remove adapters and low-quality reads. The filtered reads were aligned to the mm10 reference genome using STAR (2.7.4a) (Zhang et al., 2022). Gene expression levels were quantified using FPKM (fragments per kilobase per million reads) calculated via featureCounts (2.0.0) (Liao et al., 2014) read counts, with the FPKM >1 as a cutoff.

### ATAC-seq data analysis

Quality control and adapter trimming were performed by fastp (0.20.0) to remove replicated and low-quality reads (MAPQ <10). Trimmed reads were then aligned to the reference genome (mm10 and hg19). Peak calling for ATAC-seq was performed by the Genrich (0.6.1) ATAC-seq mode with parament “-j–p 0.01.” Peaks within the mm10 or hg19 blacklist were removed.

### ChIP-seq, CUT&Tag, and CUT&RUN data processing

Fastp (0.20.0) were used to trim adapter and quality control for ChIP-seq, CUT&Tag, and CUT&RUN reads. Trimmed sequencing reads from mouse round spermatids and E14Tg2a mESCs were aligned to the mm10 reference genome using Bowtie2 (Langmead and Salzberg, 2012) (v2.3.5.1), whereas reads from HEK293T and K562 were aligned to the hg19 reference genome. To improve the mapping accuracy, reads with an MAPQ score below 30 were excluded. PCR duplicates were removed using Sambamba (0.7.1) (Tarasov et al., 2015).

### Peak calling

#### Benchmarking of peak calling software for CUT&Tag and CUT&RUN

For the assessment of CUT&Tag/CUT&RUN peak calling methods, six algorithms were used to identify CUT&Tag/CUT&RUN peaks, including widely used algorithms for ChIP-seq MACS2 and HOMER and algorithms designed for CUT&Tag/CUT&RUN data SEACR and GoPeaks, and other two algorithms named SICER2 and Genrich were also included. Detailed paraments are as follows:

MACS2: narrow peaks for CTCF and H3K4me3 were called with parament “-f BAM/BAMPE--keep-dup all--SPMR.” Broad peaks for H3K27me3 were called with additional parament “--broad--broad-cutoff.” The threshold for peak filtering was set as q = 0.05. HOMER: findPeaks command was used with parament “-style histone-fdr 0.001” for histone modification and “-style factor-fdr 0.001” for CTCF. SICER2: peaks were identified with default paraments “-w 200-rt 1-f 150-egf 0.74-fdr 0.01-g 600-e 1000.” Genrich: peaks were called with default significance threshold p = 0.01. SEACR: the top 1% peaks were retained under the “stringent” mode. GoPeaks: peaks were called with default threshold p = 0.05. In addition, “--broad--mdist 3000” were set for H3K27me3 peak calling.

#### Consensus peak

For each protein/TF in ChIP-seq, CUT&Tag, and CUT&RUN, aligned bam files of two replicates were pooled together for peak calling using MACS2 (2.2.7.1). Narrow peaks for CTCF and H3K4me3 were called with parament “-f BAM/BAMPE--keep-dup all--SPMR.” Broad peaks for H3K27me3 were called with additional parament “--broad--broad-cutoff.”

A series of threshold values for peak filtering were set as q = 0.1, q = 0.05, q = 0.01, and q = 1e-5 (Supplementary Table S1). In addition, a loose threshold (p = 0.01) was set to detect weak signals. Since histone modifications typically occupied broad regions on genomes, sequences from −500 bp to +500 bp from the summit position of all H3K4me3 and H3K27me3 peaks were concluded. Consensus peaks were identified by merging overlapping peaks using BEDtools (v2.30.0).

## Identification of common peaks, overlap peaks, and unique peaks

We checked the overlap of consensus regions across ChIP-seq, CUT&Tag, and CUT&RUN and identified those above the peak according to the following criteria: common peaks were defined as regions simultaneously identified in ChIP-seq, CUT&Tag, and CUT&RUN with MACS2 threshold q = 0.01. OLP peaks were defined as regions identified in two of all three methods with q = 0.01 significance but could not be detected in the other method data by MACS2 (five thresholds) (Supplementary Table S2). For example, a CUT&Tag CUT&RUN OLP peak could be detected in both CUT&Tag and CUT&RUN data with an MACS2 threshold q = 0.01; however, this peak could not be detected in ChIP-seq by MACS2 with any five thresholds. A unique peak was defined as a region that was identified in one of all three methods with MACS2 threshold q = 0.01 but could not be detected in the other two methods (with any of the five thresholds).

### Fraction of reads in peaks

FRiP was calculated as the proportion of reads in all consensus regions (across the five thresholds) relative to the total mapped reads. The formula is as follows:

$$FRiP=\frac{Reads\;\;in\;\;consensus\;\;regions}{Total\;\;mapped\;\;reads}.$$

### Comparison of sensitivity

To compare the ability to detect convincingly enriched regions, OLP peaks and common peaks were defined as reliable regions, which were detectable by at least two methods with a q = 0.01 threshold. The proportions of OLP peaks and common peaks detected by each method were then compared.

### Comparison of the read distribution in common peaks

BEDtools Intersect (v2.30.0) (Quinlan and Hall, 2010) was used to identify common peaks overlapped with chromatin accessible regions (ATAC-seq peaks). For these overlapping peaks, the fraction of ChIP-seq, CUT&Tag, and CUT&RUN reads aligned to the chromatin accessible region was calculated.

### Motif analysis

Predicted CTCF binding sites in mm10 and hg19 were downloaded from the JASPAR database (Rauluseviciute et al., 2024). BEDtools was used to discover CTCF peaks within CTCF motifs.

### Hi-C data analysis

Hi-C data were processed using the HiC-Pro (3.1.0) pipeline (Servant et al., 2015) with default parameters. “hicpro2juicebox.sh” was used to convert valid read pairs into the input format required by Juicebox (Durand et al., 2016) (2.15) for visualization. Topologically associating domains (TADs) were identified with HiCExplorer (Ramírez et al., 2018).

### Quantification and statistical analysis

Sample size, mean, and significant p. values are indicated in the text, figure legends, and *Materials and methods*. Statistical analyses were performed using R (version 4.4.0) and r-based computational tools. Detailed information is provided in *Materials and methods*.

## Results

### Basis of processing and quality control of the included data

**FIGURE 2:**
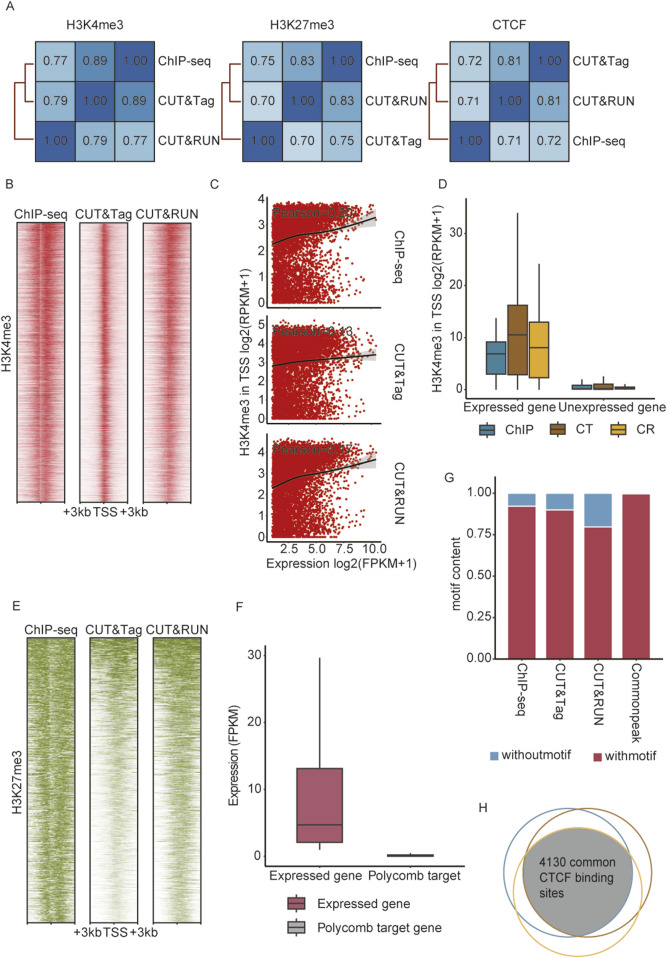
ChIP-seq, CUT&Tag, and CUT&RUN detected reliable enrichment signals in round spermatids. **(A)** Pearson correlation across peaks identified by ChIP-seq, CUT&Tag, and CUT&RUN. **(B)** Heatmaps showing H3K4me3 enrichment at the TSS of expressed genes (n = 10,083) from ChIP-seq, CUT&RUN, and CUT&Tag, with genes ordered by RPKM. **(C)** Scatterplot with a fitted curve showing the correlation between the expression level of active genes (n = 10,083) and H3K4me3 signal intensities. **(D)** Normalized H3K4me3 signal (RPKM) enrichment at promoters (TSS ±1 kb) of expressed (n = 10,083) and unexpressed (n = 19,144) RS genes. **(E)** Heatmaps showing H3K27me3 enrichment at TSS of polycomb target genes (n = 592) from ChIP-seq, CUT&RUN, and CUT&Tag. **(F)** mRNA expression (FPKM) of all expressed genes and polycomb target genes in round spermatids. **(G)** Proportions of detected peaks containing the CTCF motif across ChIP-seq, CUT&RUN, and CUT&Tag. **(H)** Venn diagram illustrating the overlap of CTCF motif-containing peaks identified by ChIP-seq, CUT&RUN, and CUT&Tag.

CUT&Tag and CUT&RUN experiments were performed following established protocols (Skene and Henikoff, 2017; Kaya-Okur et al., 2019), with two replicates per group. All included ChIP-seq, CUT&Tag, and CUT&RUN data were preprocessed with recommended pipelines (Skene and Henikoff, 2017; Kaya-Okur et al., 2019; Nakato and Sakata, 2021). Correlation analysis across three techniques confirmed the data consistency; meanwhile, the signal distributions of H3K4me3, H3K27me3, and CTCF between replicates exhibited a high concordance (Figure 2A; Supplementary Figure S1). Replicates of each group were pooled for downstream peak calling and statistical analyses at the optimal sequencing depth. As the algorithms can influence peak identification, we compared the performance of different peak callers, focusing on two key factors: peak numbers and motif correspondence (Supplementary Figures S2, S3). MACS2 was selected as the peak caller for subsequent analyses. Antibodies used for CUT&Tag and CUT&RUN were carefully chosen based on the proven effectiveness in these specific techniques (Franklin et al., 2022; Michelatti et al., 2024; Pal et al., 2023; Chrysanthou et al., 2022). However, the comparative ChIP-seq datasets (e.g., H3K4me3) employed antibodies only validated for conventional ChIP. To address potential biases arising from antibody differences, we implemented quality control (Supplementary Figure S4). Both epitope analysis and enrichment correlation analysis showed high reproducibility (r = 0.88, 0.91, and 0.95), demonstrating minimal technical bias from antibody differences.

### Validation of ChIP-seq, CUT&Tag, and CUT&RUN for reliable detection of histone modifications and transcription factor binding

H3K4me3 is a histone modification marker predominantly associated with gene promoters, typically enriched near transcription start sites (TSS), marking genes in an active transcriptional activity (Millán-Zambrano et al., 2022). To evaluate the performances of ChIP-seq, CUT&Tag, and CUT&RUN in detecting active histone modifications, we analyzed the correlation between H3K4me3 signal intensities and mRNA expression levels in round spermatids. Among the 10,083 genes identified by mRNA sequencing, H3K4me3 signals were predominantly enriched in their promoter regions (Figure 2B) and showed a strong positive correlation with gene expression, as indicated by Pearson correlation coefficients (Figure 2C). In contrast, none of the methods detected H3K4me3 signals in the promoter of inactive genes (Figure 2D). These indicated the high sensitivity and reliability of ChIP-seq, CUT&Tag, and CUT&RUN in profiling histone modifications associated with active transcription, highlighting their robustness for profiling epigenetic markers linked to gene activation. Meanwhile, H3K27 trimethylation, catalyzed by polycomb repressive complex 2 (PRC2), serves as a well-established marker of gene temporal silencing. During spermatogenesis, PRC2-targeted genes remain relatively conserved (Erkek et al., 2013). The H3K27me3 patterns in these regions, identified by ChIP-seq, CUT&Tag, and CUT&RUN, were consistent with prior knowledge, and none of these regions were detected with mRNA expression (Figures 2E,F). CTCF is a pivotal chromatin architecture protein essential for the establishment and maintenance of higher-order chromatin structure (Oudelaar and Higgs, 2021). Studies have shown that CTCF collaborates with lineage-specific pioneer transcription factors to establish chromatin interaction hubs (Liu et al., 2023b). Genomic locations of CTCF detected by three techniques were validated against predicted CTCF binding sites from the JASPAR database. Over 75% of the CTCF peaks aligned with CTCF motifs (Figure 2G). Furthermore, 4,130 peaks within CTCF binding sites were identified simultaneously by all three techniques, providing preliminary evidence of their reliability and efficiency in detecting general transcription factors (Figure 2H).

### Impact of peak filtration criteria on enrichment quantification and signal specificity comparisons

**FIGURE 3:**
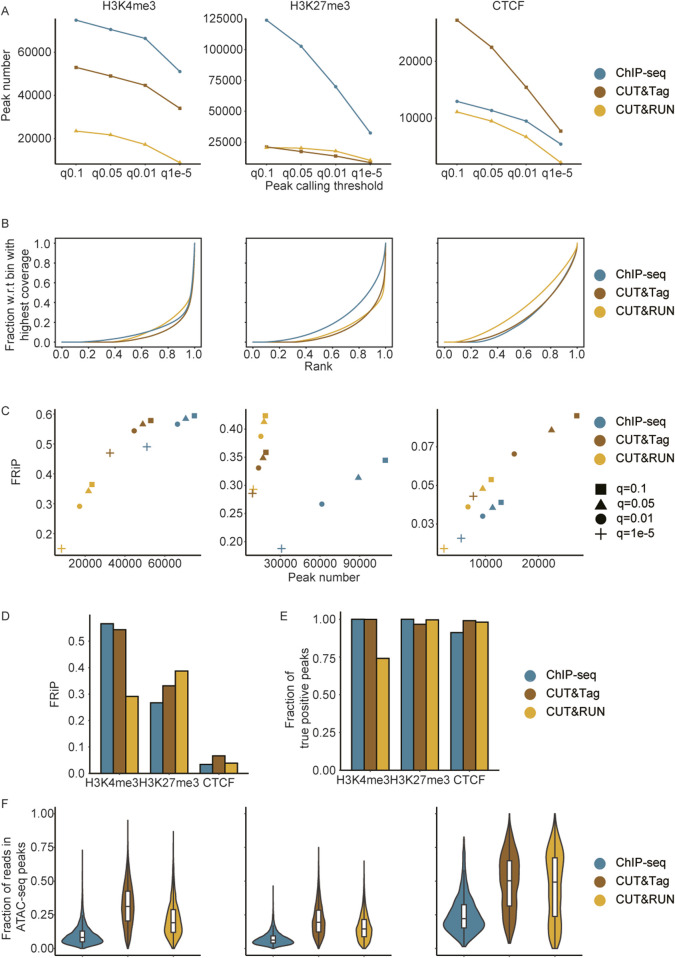
Impact of peak filtration criteria on enrichment quantification and signal specificity comparisons. **(A)** Comparison of H3K4me3, H3K27me3, and CTCF consensus peaks identified using different thresholds across methods. **(B)** Cumulative curves illustrating the enrichment specificity of H3K4me3, H3K27me3, and CTCF across methods. **(C)** Scatterplot showing the relationship between peak numbers and FRiP scores for H3K4me3, H3K27me3, and CTCF peaks, called at various MACS2 thresholds, with comparisons based on consensus peaks. **(D)** FRiP scores across methods for CTCF, H3K27me3, and H3K4me3, based on consensus peaks called at the MACS2 q = 0.01 threshold. **(E)** Comparison of sensitivity (fraction of true positive peaks) across methods for CTCF, H3K27me3, and H3K4me3, with true positive peaks defined as those detected by at least two methods at the MACS2 q = 0.01 threshold. **(F)** Comparison of the read distribution (fraction of H3K4me3, H3K27me3, and CTCF reads in accessible chromatin overlapped with common peaks).

The criteria used for peak filtration significantly impact the quantification of signal intensity in enrichment sites. Therefore, establishing appropriate peak filtering standards is crucial for evaluating the strengths or limitations of these three techniques. We systematically compared the peak detection efficiency among ChIP-seq, CUT&Tag, and CUT&RUN under varying thresholds. The analysis revealed that for histone modifications, ChIP-seq detected the largest number of peaks, whereas CUT&Tag outperformed the other methods in detecting general TFs (Figure 3A). With increasingly stringent thresholds, the number of detected peaks gradually decreased. Notably, the number of peaks of H3K4me3 declined sharply between q = 0.01 and q = 1e-5, while the H3K27me3 peaks detected by ChIP-seq experienced a significant decrease from q = 0.05 to q = 0.01. For CTCF, the most substantial reduction in peak numbers across all three methods was observed during the transition from q = 0.05 to q = 0.01 (Figure 3A).

Typically, differences in library preparation led to variations in background noise levels (Diaz et al., 2012; Ramírez et al., 2014). We first analyzed cumulative read coverage profiles (Figure 3B). CUT&Tag demonstrated superior overall performance in signal enrichment for H3K4me3 and CTCF, as evident from the steeper curve, which indicated a greater fraction of enriched signals concentrated in the highest-coverage bins, highlighting its higher specificity and lower background noise. CUT&RUN showed comparable performance to that of CUT&Tag in detecting H3K27me3 but was significantly less effective than both ChIP-seq and CUT&Tag in identifying general transcription factors. Additionally, CUT&Tag exhibited specific enrichment in diploid cells, further underscoring its effectiveness (Supplementary Figure S5B).

The fractions of reads in peaks (FRiP) across different thresholds (q = 0.1, q = 0.05, q = 0.01, and q = 1e-5) were also compared (Figure 3C). For H3Kme3, both CUT&Tag and ChIP-seq detected sufficient peak numbers with high signal-to-noise ratios, while CUT&RUN performed worse than the former two in terms of peak number and FRiP. In terms of H3K27me3, ChIP-seq showed a relatively low signal-to-noise ratio, whereas CUT&RUN and CUT&Tag achieved comparable peak numbers with higher signal-to-noise ratios. Regarding CTCF, CUT&Tag outperformed the other two methods in terms of peak numbers and FRiP, aligning with similar findings observed in diploid cells (Figure 3C; Supplementary Figures S5B, C). Based on the above comparisons, we took a q-value threshold of 0.01 as the significance standard for peak calling in subsequent analyses. The average FRiP level of CTCF was lower than that of H3K4me3 and H3K27me3 (Figure 3D), which could be attributed to the fact that CTCF is a transcription factor with specific and narrower binding sites, whereas H3K4me3 and H3K27me3 are histone modifications associated with broader chromatin regions, resulting in a higher overall FRiP score.

### Sensitivity and chromatin accessibility bias in ChIP-seq, CUT&Tag, and CUT&RUN

Serving as the main methods for detecting protein and transcription factor binding signals, ChIP-seq, CUT&Tag, and CUT&RUN face challenges in identifying genome-wide reference sets or established marker sets to validate the sensitivity of the detected signals. Moreover, conducting genome-wide validation experiments remains a substantial hurdle. To address this, we defined peaks identified by at least two methods as “True Positive Peaks (TPPs),” which were used as control sets in subsequent sensitivity comparisons. ChIP-seq and CUT&Tag demonstrated near-complete sensitivity in detecting reliable H3K4me3 binding information (0.99), whereas CUT&RUN showed lower sensitivity for H3K4me3 detection (0.74). For H3K27me3 and CTCF, all three methods detected the most reliable enrichment sites (>0.9) (Figure 3E). These results indicated that all three methods exhibited high sensitivity in identifying activation and repressive histone modifications, as well as true transcription factor enrichment regions, in both haploid and diploid cells (Supplementary Figure S5D). The chromatin accessibility landscape (ATAC-seq) enabled us to figure out the potential enrichment preference to accessible DNA. By analyzing the proportion of reads from common peaks associated with chromatin open sites, we found that signals in CUT&Tag and CUT&RUN were more enriched in accessible chromatin compared to those in ChIP-seq. These findings suggest that CUT&Tag and CUT&RUN may exhibit a bias toward more accessible chromatin (Figure 3F; Supplementary Figure S5E).

### CUT&Tag and CUT&RUN detected novel CTCF binding sites in round spermatids

**FIGURE 4:**
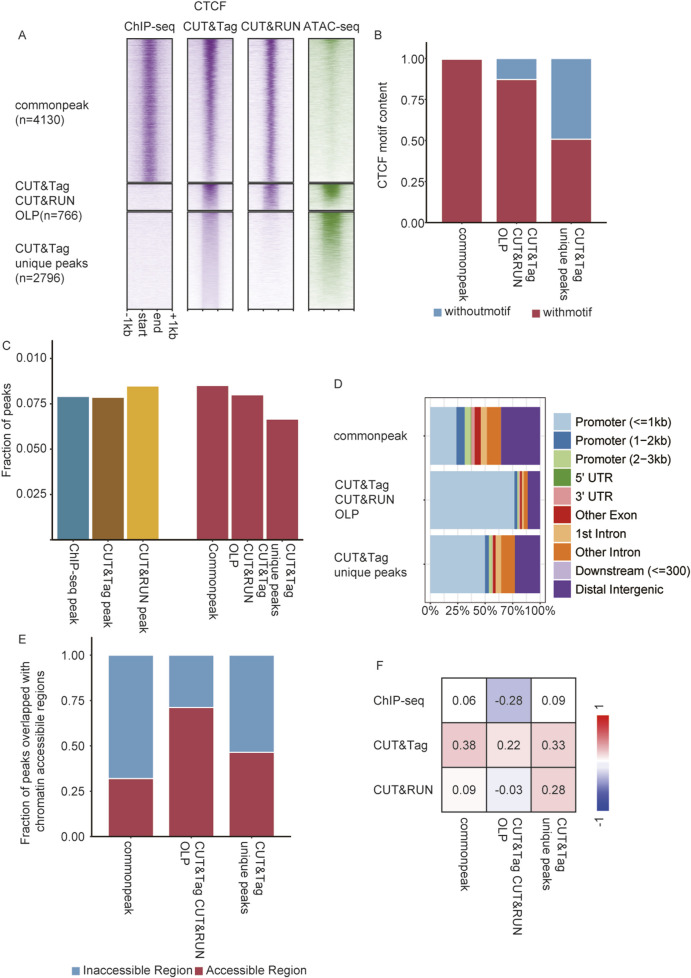
CUT&Tag and CUT&RUN detected novel CTCF binding sites associated with accessible chromatin. **(A)** Heatmap showing ChIP-seq, CUT&Tag, and CUT&RUN enrichment and chromatin accessibility (ATAC-seq) at CTCF common peak, CT&CR OLPs, and CT unique peaks. **(B)** CTCF motif content in common peak, CT CR OLPs, and CT unique peaks. **(C)** Left: fraction of consensus peaks located in TAD boundaries from ChIP-seq, CUT&Tag, and CUT&RUN. Right: fraction of the common peak, CT CR OLPs, and CT unique peaks located in TAD boundaries. **(D)** Genomic distributions of common peaks, CT CR OLPs, and CT unique peaks. **(E)** Fraction of peaks overlapping with chromatin-accessible regions. Left: all CTCF peaks detected by ChIP-seq, CUT&Tag, and CUT&RUN. Right: CTCF common peaks, CT CR OLPs, and CT unique peaks. **(F)** Point-biserial correlation between CTCF enrichment and chromatin-accessible regions.

Remarkably, CUT&Tag detected exceptionally more CTCF peaks than ChIP-seq, while maintaining a comparable high signal-to-noise ratio (Figure 3A). Given the differing performance of these techniques in detecting TFs versus histone modifications, we further analyzed the differential CTCF occupancy detected by all three techniques. A total of 766 CTCF binding sites were absent in ChIP-seq results but were detected by both CUT&Tag and CUT&RUN, which we referred to as overlap peaks (OLPs) (*Methods Peak Calling* Section). Additionally, 2,796 CTCF sites were uniquely detected by CUT&Tag, which were termed CT unique peak (Figure 4A). Motif enrichment analysis revealed that 99% of common peaks contained CTCF motifs (4,113/4,130), while 87% of OLPs (668/766) and 50% of CT unique peaks contained CTCF motifs (1,420/2,796) (Figure 4B). This indicated that novel peaks could be identified by CUT&Tag methods. As a chromatin architectural protein that mediates loop extrusion, CTCF has been reported to be enriched at promoter regions and the boundaries of topological associating domains (TAD boundaries) (Davidson et al., 2023). Using Hi-C data from round spermatids (Luo et al., 2020), novel CTCF peaks identified by CUT&Tag were further investigated to determine their association with TAD boundaries. CT unique peaks displayed a proportional distribution at TAD boundaries like the overall peak population (Figure 4C). A total of 2,389 TAD boundaries were identified in round spermatids. A consistent proportion of CTCF peaks were located at TAD boundaries across the methods, with 7.9% being detected by ChIP-seq, 7.6% by CUT&Tag, and 8.5% by CUT&RUN (Figure 4C). Of the 2,389 TAD boundaries analyzed, 536 (22%) contained CTCF peaks identified by ChIP-seq, 666 (28%) contained peaks identified by CUT&Tag, and 373 (16%) contained peaks identified by CUT&RUN. Additionally, 11% (276) of the TAD boundaries contained CTCF common peaks detected by all three techniques (Supplementary Figure S7). Genomic distribution analysis indicated that the majority of common CTCF peaks were located in distal non-coding intergenic regions, with 37% of them mapped to promoter regions associated with 1,531 genes. Conversely, 80% of OLPs were localized to promoter regions corresponding to 619 genes, while 56% of CT unique peaks were situated in the promoter regions of 1,575 genes (Figure 4D).

### CUT&Tag exhibited a higher sensitivity for detecting peaks in accessible chromatin

Since most CTCF sites absent in ChIP-seq were located at active transcription sites, it raised the question of whether chromatin accessibility affected the detection of these peaks identified by CUT&Tag and CUT&RUN. To explore this, the overlap of CTCF peaks, including common peaks, OLPs, and CT unique peaks, with accessible chromatin (ATAC-seq peaks) was analyzed. Approximately 31% of the common peaks overlapped with accessible chromatin regions, while 71% of OLPs and 46% of CT unique peaks were found in accessible chromatin (Figure 4E). Point-biserial correlation analysis was performed to assess the relationship between the CTCF enrichment detected by the three methods and the location of peaks within accessible chromatin. It was shown that the CTCF signal intensity detected by ChIP-seq exhibited little correlation or was negatively correlated with open chromatin regions. For CUT&RUN, CTCF signals in common peaks and OLPs were also uncorrelated with chromatin accessibility, while signals from CUT&Tag unique peaks showed a weak positive correlation. Notably, CTCF signal intensity detected by CUT&Tag across all peak types displayed a weak positive correlation with accessible chromatin regions (Figure 4F; Supplementary Figure S6). CUT&Tag exhibited higher affinity for binding signals in accessible chromatin. This characteristic allowed CUT&Tag to achieve higher resolution in detecting complicated binding patterns within open chromatin regions, effectively mitigating the influence of the signal-to-noise ratio. These peak types were further categorized into nine groups based on their overlap with motifs. Genomic distribution analysis revealed that the novel peaks detected by CUT&Tag were primarily enriched in promoter regions, consistent with the proportion of peaks located within accessible chromatin regions. Additionally, correlation analysis validated the authenticity of motifs in identifying true peaks (Supplementary Figures S8A–C).

### Enhanced CUT&Tag sensitivity in accessible chromatin demonstrated in diploid cells

The above conclusion prompted an inquiry into the extent to which chromatin accessibility may affect the performance of CUT&Tag in detecting protein–DNA interactions in diploid cells. To delve deeper into this relationship, the same analysis was conducted in haploid cells.

**FIGURE 5:**
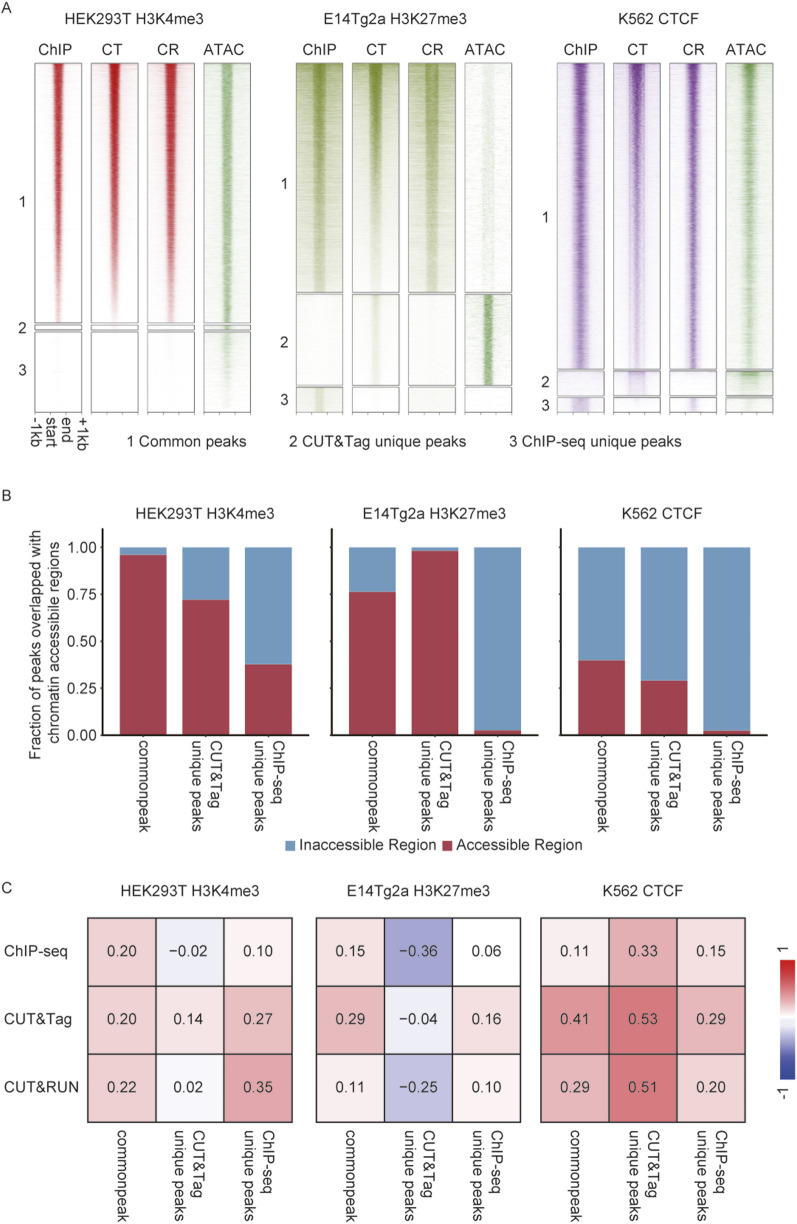
Comprehensive analysis of chromatin accessibility bias in CUT&Tag in diploid cells. **(A)** Heatmap showing ChIP-seq, CUT&Tag, and CUT&RUN enrichment, alongside chromatin accessibility (ATAC-seq) for common peak, CT unique peaks, and ChIP-seq unique peaks. **(B)** Fraction of peaks overlapped with chromatin-accessible regions. **(C)** Point-biserial correlation between chromatin accessibility and enrichment of H3K27me3 in E14Tg2a cells, H3K4me3 in HEK293T cells, and CTCF in K562 cells.

For H3K27me3 in E14Tg2a cells, 6,869 common peaks were detected, alongside 2,727 CUT&Tag unique peaks and 750 ChIP-seq unique peaks. Strong H3K27me3 enrichment was observed in the common peaks for both methods, whereas unique peaks displayed weaker signals, with CUT&Tag showing the least enrichment (Figure 5A). A similar pattern was observed for H3K4me3 in HEK293T cells, where 12,712 common peaks were detected. However, only 257 peaks were unique to CUT&Tag, compared to 3,929 unique to ChIP-seq, with both sets of unique peaks exhibiting weaker signals. For CTCF in K562 cells, 12,067 common peaks were identified, along with 972 CUT&Tag-specific peaks and 578 ChIP-seq-specific peaks. Analysis of chromatin accessibility revealed a pronounced preference for accessible regions by CUT&Tag. Among CUT&Tag-specific peaks, 92% of H3K27me3 peaks, 37% of H3K4me3 peaks, and 29% of CTCF peaks overlapped with accessible chromatin regions, compared to the minimal overlap observed for ChIP-seq-specific peaks. In contrast, 31% of common peaks overlapped with accessible chromatin regions, indicating that shared peaks were less biased toward open chromatin (Figure 5B).

Correlation analysis between signal intensity and chromatin accessibility reveals distinct patterns across methods. For H3K27me3, correlations were generally low, consistent with its repressive nature. The highest correlation for common peaks was observed in CUT&RUN (r = 0.22), while CUT&Tag showed a moderate correlation (r = 0.14) for its unique peaks, which was higher than that of ChIP-seq (r = −0.02). For H3K4me3, correlations were stronger, reflecting its role in active transcription. CUT&Tag demonstrated the highest correlation at its unique peaks (r = 0.53) compared to CUT&RUN (r = 0.51) and ChIP-seq (r = 0.33). Across common peaks, CUT&Tag also exhibited higher correlations (r = 0.41) compared to CUT&RUN (r = 0.29) and ChIP-seq (r = 0.11). For CTCF, all three methods showed relatively strong correlations with chromatin accessibility, particularly at CUT&Tag unique peaks (r = 0.53) and CUT&RUN unique peaks (r = 0.51). Correlations at common peaks were strongest for CUT&Tag (r = 0.41), with lower values observed for CUT&RUN (r = 0.29) and ChIP-seq (r = 0.11). These results highlighted CUT&Tag’s superior sensitivity in detecting signals within open chromatin regions, particularly for H3K4me3 and CTCF (Figure 5C).

## Discussion

Our systematic comparison of three widely utilized chromatin–protein interaction profiling techniques—ChIP-seq, CUT&RUN, and CUT&Tag—revealed fundamental differences in their performance of authentic peak detection, signal detection sensitivity, and sequence biases arising from fragmentation specificity. As a fundamental verification of the previous research reported, we demonstrated that while all three techniques reliably identify canonical binding sites, they exhibited marked variations in the signal-to-noise ratio. CUT&Tag had a higher resolution and reduced background noise compared to ChIP-seq and CUT&RUN, which was consistent with the findings of previous studies that highlighted the advantages of enzymatic fragmentation over sonication (Kaya-Okur et al., 2019; Kaya-Okur et al., 2020).

Results also indicated that CUT&Tag and CUT&RUN exhibit higher sensitivity than ChIP-seq in detecting active histone modifications such as H3K4me3, which is consistent with our findings that these methods are well-suited for profiling active chromatin. However, both methods exhibit reduced efficiency in identifying repressive modifications such as H3K27me3, which are typically associated with compact, transcriptionally silent chromatin. Their reliance on enzymatic accessibility limits their ability to capture signals from closed chromatin regions. In contrast, ChIP-seq employs formaldehyde crosslinking and sonication-based fragmentation, enabling more effective recovery of protein–DNA complexes from condensed chromatin and, thus, more reliable detection of repressive histone markers. Accordingly, CUT&RUN and CUT&Tag were employed for the detection of active transcriptional markers, while ChIP-seq was preferred for profiling repressive histone modifications, given its superior performance in compact chromatin regions.

Our analysis identified novel CTCF binding sites in round spermatids that were not detected by ChIP-seq. These sites were predominantly located in promoters of actively transcribed genes and contained canonical CTCF motifs. While both CUT&Tag and CUT&RUN detected these peaks, we noted important methodological considerations regarding potential false positives. The Tn5 transposase employed in CUT&Tag exhibits a well-documented preference for open chromatin regions. Consequently, the observed signal intensities may reflect chromatin accessibility biases rather than true protein occupancy. This bias likely explains the enrichment of CUT&Tag-specific CTCF peaks in promoter regions, which typically maintain accessible chromatin configurations. In contrast, CUT&RUN utilizes MNase for targeted cleavage and demonstrates less pronounced accessibility bias, potentially providing a more accurate representation of protein–DNA interactions. These findings emphasize the need for cautious interpretation of CUT&Tag data, particularly when analyzing transcriptionally active regions where chromatin accessibility and protein binding are inherently correlated.

These considerations are particularly crucial when studying transcription factor dynamics or chromatin modifications, where the methodological precision is key. Additionally, the potential biases in sequencing read counts introduced by allele-specific binding are also considered. We used paternal haploid reproductive cells as the research model, which contained only a single copy of each chromosome and were unable to be cultured or passaged, preventing potential abnormal differentiation and minimizing batch-to-batch variability. In addition to this, Matsuwaka et al. (2025) reported the different deposition of H3K27me2/3 and H3K36me3 between paternal and maternal chromosomes in gametes—a phenomenon that may also occur in diploid cell lines. Such an allelic imbalance in TF occupancy can lead to misinterpretation of peak signals by peak callers, resulting in statistical biases in peak detection. By utilizing haploid cells, we circumvent these confounding factors, enabling a more accurate and unbiased assessment of the true performance of ChIP-seq, CUT&RUN, and CUT&Tag.

Beyond methodological comparisons, our findings provide substantive biological insights for developmental and reproductive research works. The identification of novel CTCF binding sites in round spermatids expands the catalog of regulatory elements in male germ cells. These sites harbor unique motif variants that may contribute to stage-specific chromatin organization during spermatogenesis. Together with our curated datasets, these results not only establish a reference map for spermatid epigenomics but also offer a framework for selecting appropriate profiling strategies based on the biological context. All datasets are publicly available to support future investigation of the 3D chromatin architecture during spermatogenesis.

Despite the comprehensive design and systematic evaluation presented in this study, several limitations should be acknowledged. We concentrated on a restricted subset of histone modifications and transcription factors, thereby limiting the generalizability of our findings to the broader range of chromatin features. Despite efforts to standardize experimental conditions, variations in antibody quality and enzymatic activity across the different methods could still have contributed to observed differences in peak detection. Furthermore, while the open chromatin bias inherent to CUT&Tag was recognized, further investigations are required to comprehensively assess how this bias may influence the interpretation of chromatin landscapes in different genomic contexts. Although our validation (Supplementary Figure S4) showed a similar antibody performance, differences in antibodies may still lead to potential bias. Cross-method comparisons should be interpreted with caution.

In summary, CUT&Tag and CUT&RUN demonstrate clear advantages in the resolution and signal-to-noise ratio, making them particularly effective for profiling transcription factors and active histone modifications. However, their reliance on enzymatic cleavage introduces a bias toward accessible chromatin, limiting their utility in detecting repressive markers such as H3K27me3. In contrast, ChIP-seq, with its crosslinking and sonication-based approach, remains more suitable for profiling compact, transcriptionally silent chromatin. Given these distinctions, the choice of the method should be guided by the chromatin context, nature of the target protein, and specific experimental goals. Our study offers a comprehensive benchmark for method selection, providing practical guidance for future chromatin–protein interaction studies across diverse regulatory landscapes.

## Data Availability

The datasets presented in this study can be found in online repositories. The names of the repository/repositories and accession number(s) can be found in the article/Supplementary Material.

## Supplementary material

SUPPLEMENTARY FIGURE S1 Correlation analysis of replicates across ChIP-seq, CUT&Tag, and CUT&RUN for detecting H3K4me3, H3K27me3, and CTCF enrichment in round spermatids.

SUPPLEMENTARY FIGURE S2 Comparison of performance across peak calling algorithms. **(A)** Number of peaks identified by MACS2, HOMER, SICER2, Genrich, SEACR, and GoPeaks. **(B)**
Intersection of CUT&Tag and CUT&RUN peaks identified by peak calling algorithms. **(C)** Distribution of peak length identified by peak calling algorithms.

SUPPLEMENTARY FIGURE S3 Comparison of performance in H3K27me3 detection across peak calling algorithms. **(A)** Density distribution of the peak number overlapped with H3K27me3 and CTCF peaks identified by different peak callers. **(B)** Density distribution of the gene number overlapped with H3K27me3 peaks identified by different peak callers. **(C)** Distribution of the motif distance to peak center identified by peak calling algorithms.

SUPPLEMENTARY FIGURE S4 Validation of antibodies targeting H3K4 trimethylation used in this study. **(A)** Epitope and antigenic sequence similarity between antibodies. **(B)** Correlation of peaks generated by different H3K4me3 antibodies across datasets (GSE49847, GSE61248, GSE210815, GSE150254, and GSE54955).

SUPPLEMENTARY FIGURE S5 Benchmarking of ChIP-seq, CUT&Tag, and CUT&RUN in diploid cell lines. **(A)** Correlation analysis across ChIP-seq, CUT&Tag, and CUT&RUN. **(B)** Cumulative curve illustrating enrichment specificity for H3K4me3 in HEK293T cells, H3K27me3 in E14Tg2a cells, and CTCF in K562 cells across ChIP-seq, CUT&Tag, and CUT&RUN. **(C)** Scatterplot showing the relationship between peak number and FRiP of HEK293T cells, H3K27me3 in E14Tg2a cells, and CTCF in K562 cells under different MACS2 thresholds; a comparison was based on consensus peaks. **(D)** Comparison of the sensitivity (fraction of OLPs) across methods for CTCF, H3K27me3, and H3K4me3; comparison was based on consensus peaks. **(E)** Comparison of the read distribution (fraction of H3K4me3, H3K27me3, and CTCF reads in accessible chromatin overlapped with common peaks).

SUPPLEMENTARY FIGURE S6 Point-biserial correlation between signal intensity and chromatin accessibility. **(A)** Round spermatids; **(B)** K562 cell line.

SUPPLEMENTARY FIGURE S7 3D chromatin pattern validated CTCF distribution across three techniques. **(A)** Fraction of CTCF located in TAD boundaries. **(B)** Examples of the overlap between TAD boundaries and CTCF binding sites, alongside the diagram comparing the differences in peaks identified by ChIP-seq, CUT&Tag, and CUT&RUN.

SUPPLEMENTARY FIGURE S8 Common peaks, CT CR OLPs, and CT unique peaks were classified based on whether they contained CTCF motifs. **(A)** Genomic distributions of common peaks, CT CR OLPs, and CT unique peaks. **(B)** Fraction of peaks overlapped with chromatin accessible regions. **(C)** Comparison of the expression of genes whose promoters overlapped with common peaks, CT CR OLPs, and CT unique peaks. **(D)** Point-biserial correlation between CTCF enrichment and chromatin accessible regions.

SUPPLEMENTARY FIGURE S9 Immunofluorescence staining and purity assessment of round spermatids. Immunofluorescence staining of cell suspension from counterflow centrifugal elutriation experiments demonstrating the purity of round spermatids. Cells were stained with PNA, and nuclei were counterstained with DAPI. Images were captured using a confocal microscope at ×40 and ×60 magnifications, with three replicates for each magnification.
