# Supplementary figures and images for: Benchmark of chromatin–protein interaction methods in haploid round spermatids
Source: Front Cell Dev Biol. 2025 May 13;13:1572405. doi: 10.3389/fcell.2025.1572405 (PMC12106302; doi:10.3389/fcell.2025.1572405)

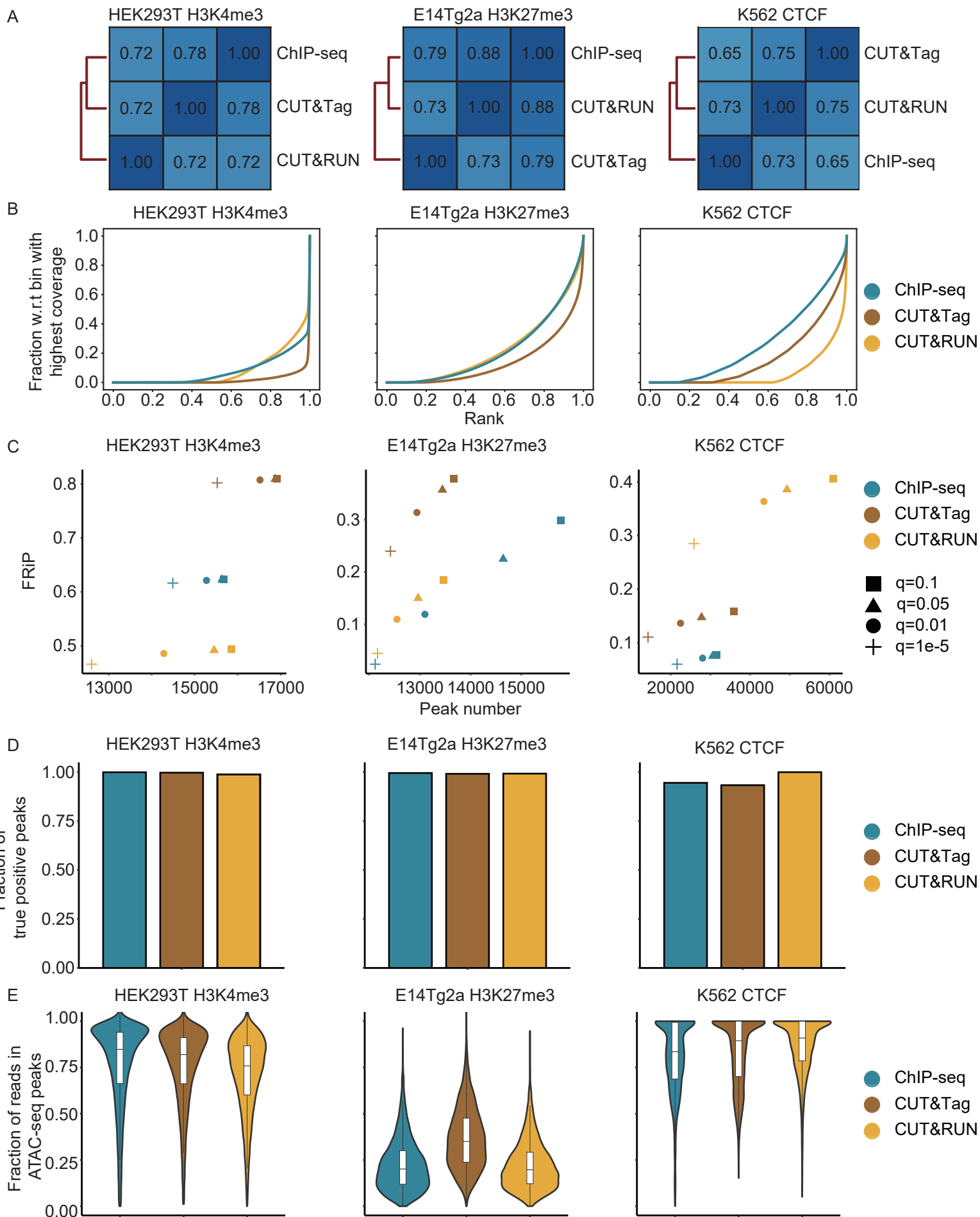

Supplement: Supplementary file 1 [file Image5.pdf]

40 X

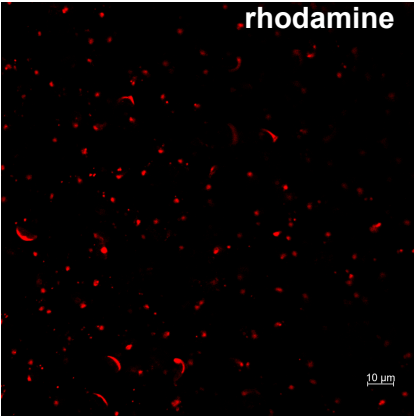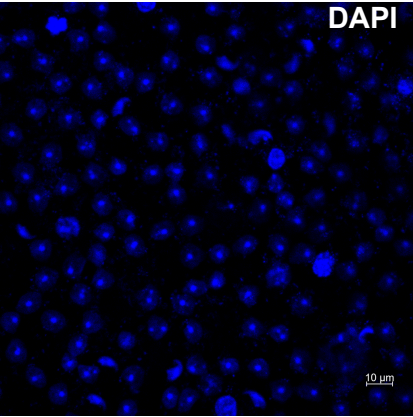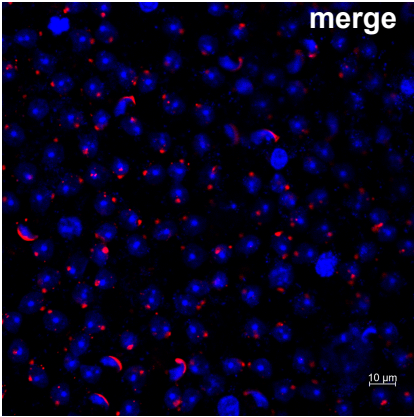

60 X

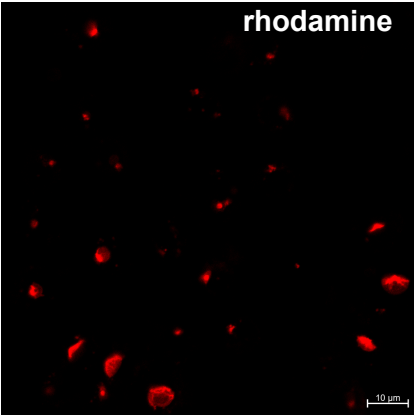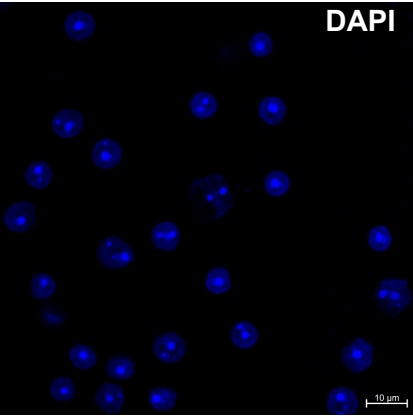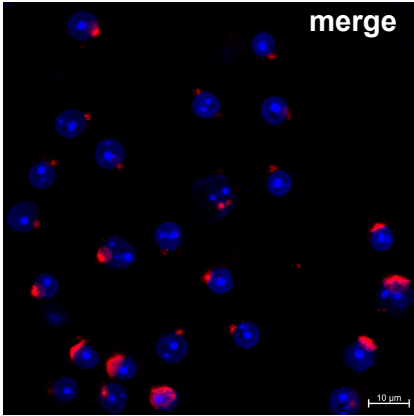

Supplement: Supplementary file 3 [file Image9.pdf]

A

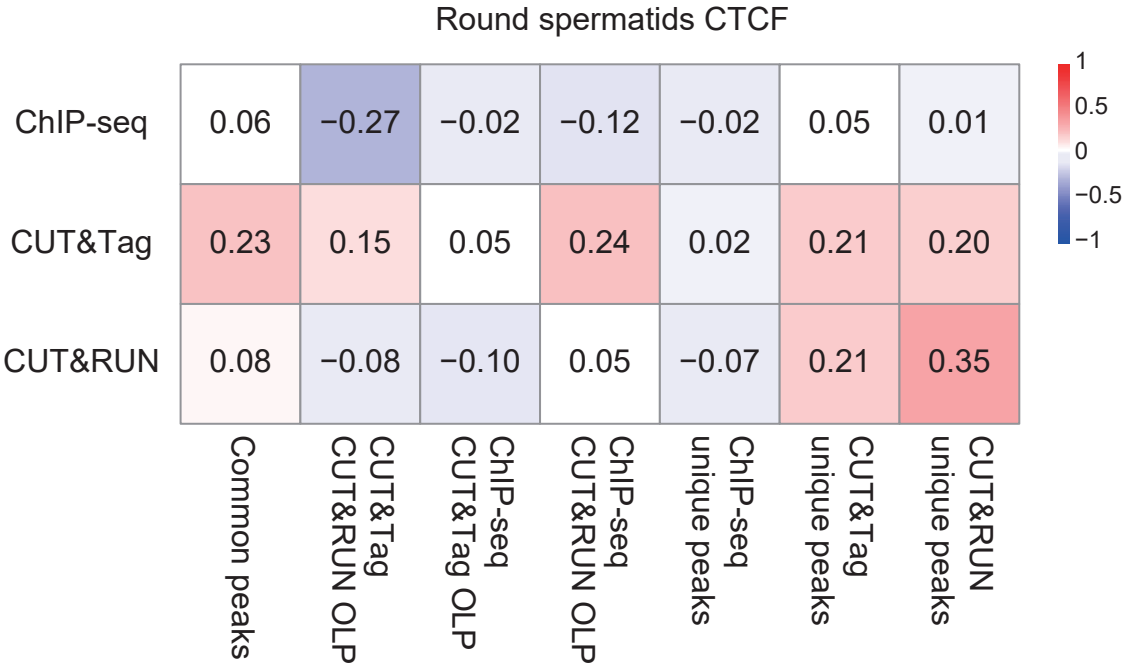

B

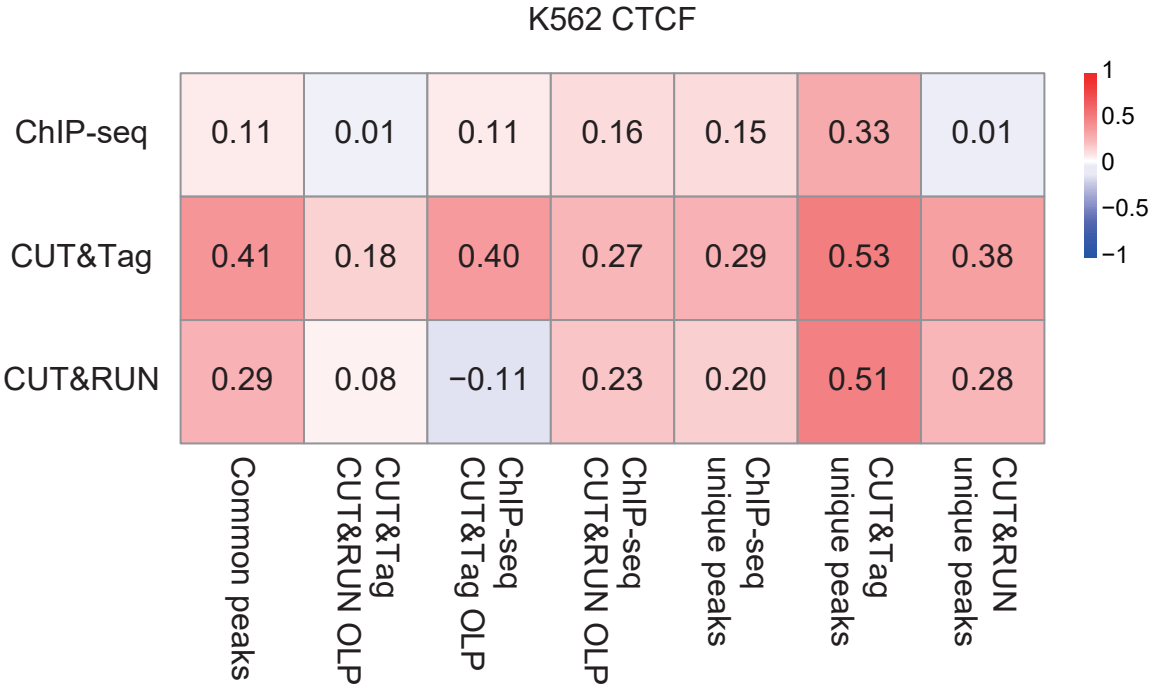

Supplement: Supplementary file 4 [file Image6.pdf]

A

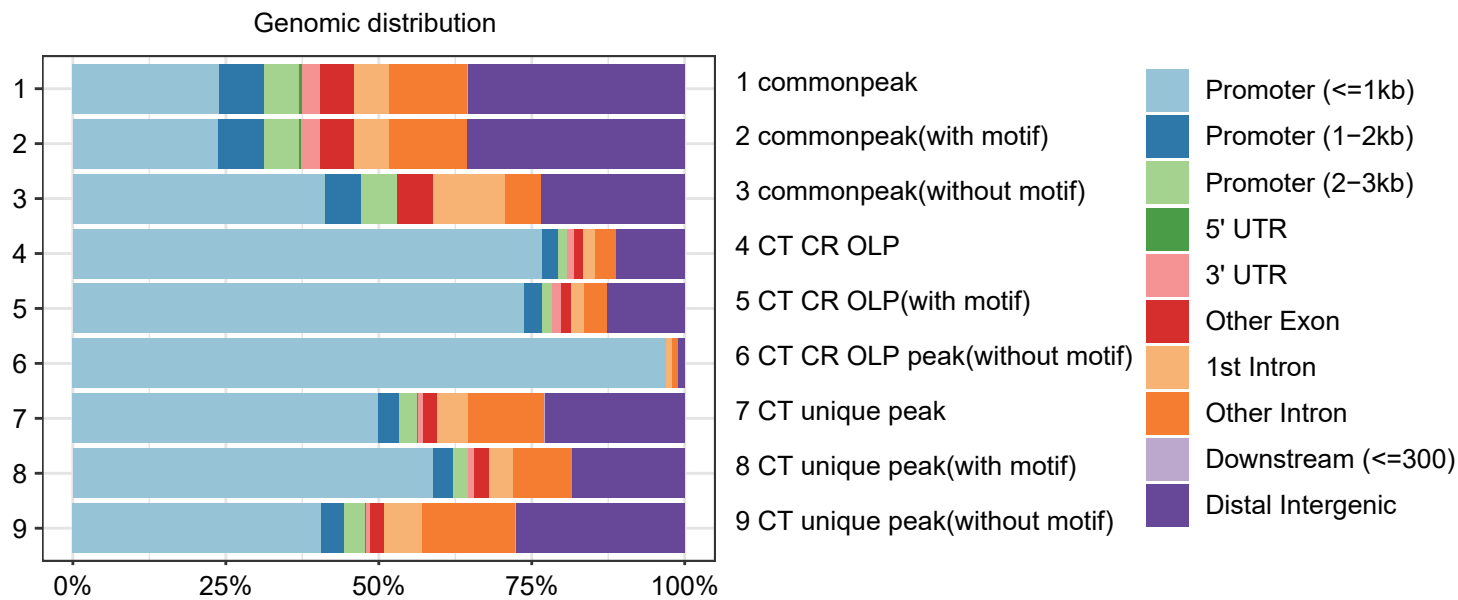

B

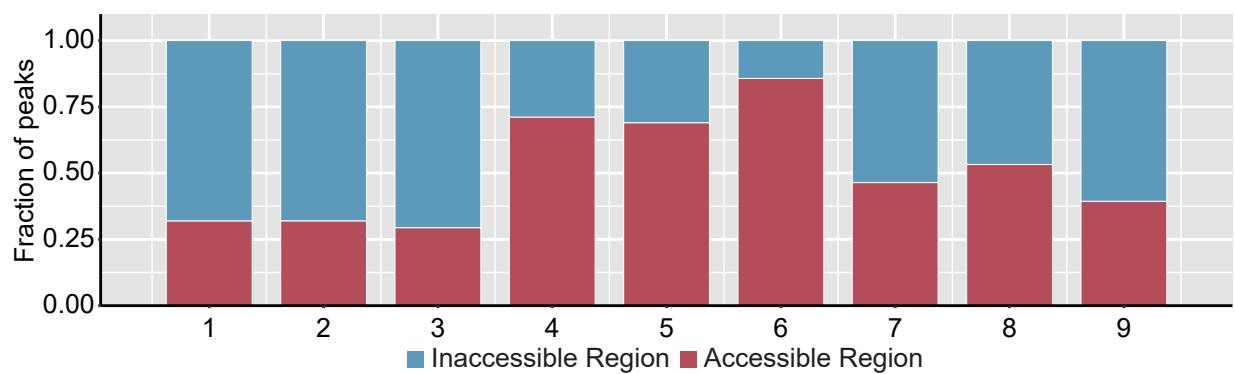

C

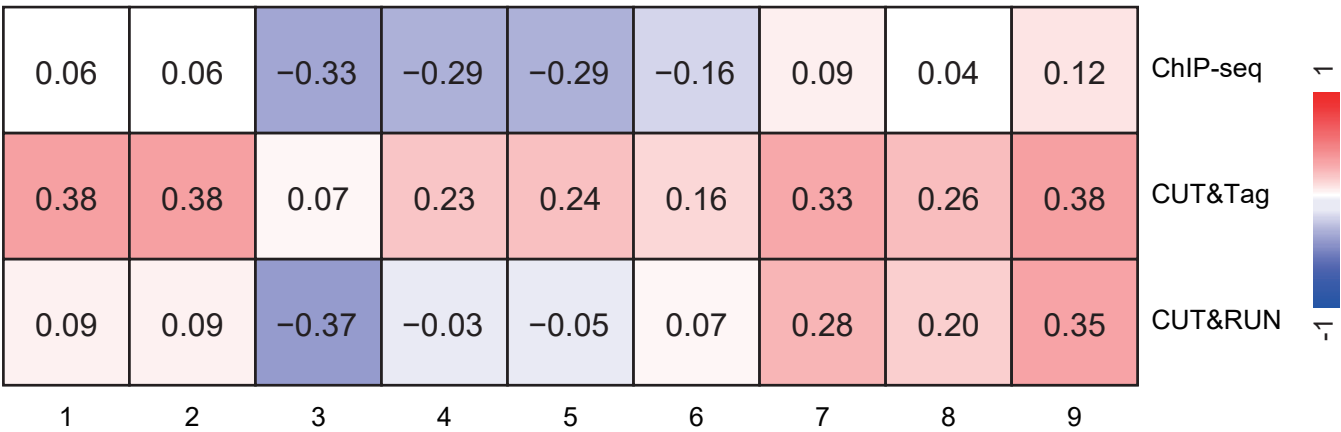

Supplement: Supplementary file 5 [file Image8.pdf]

A

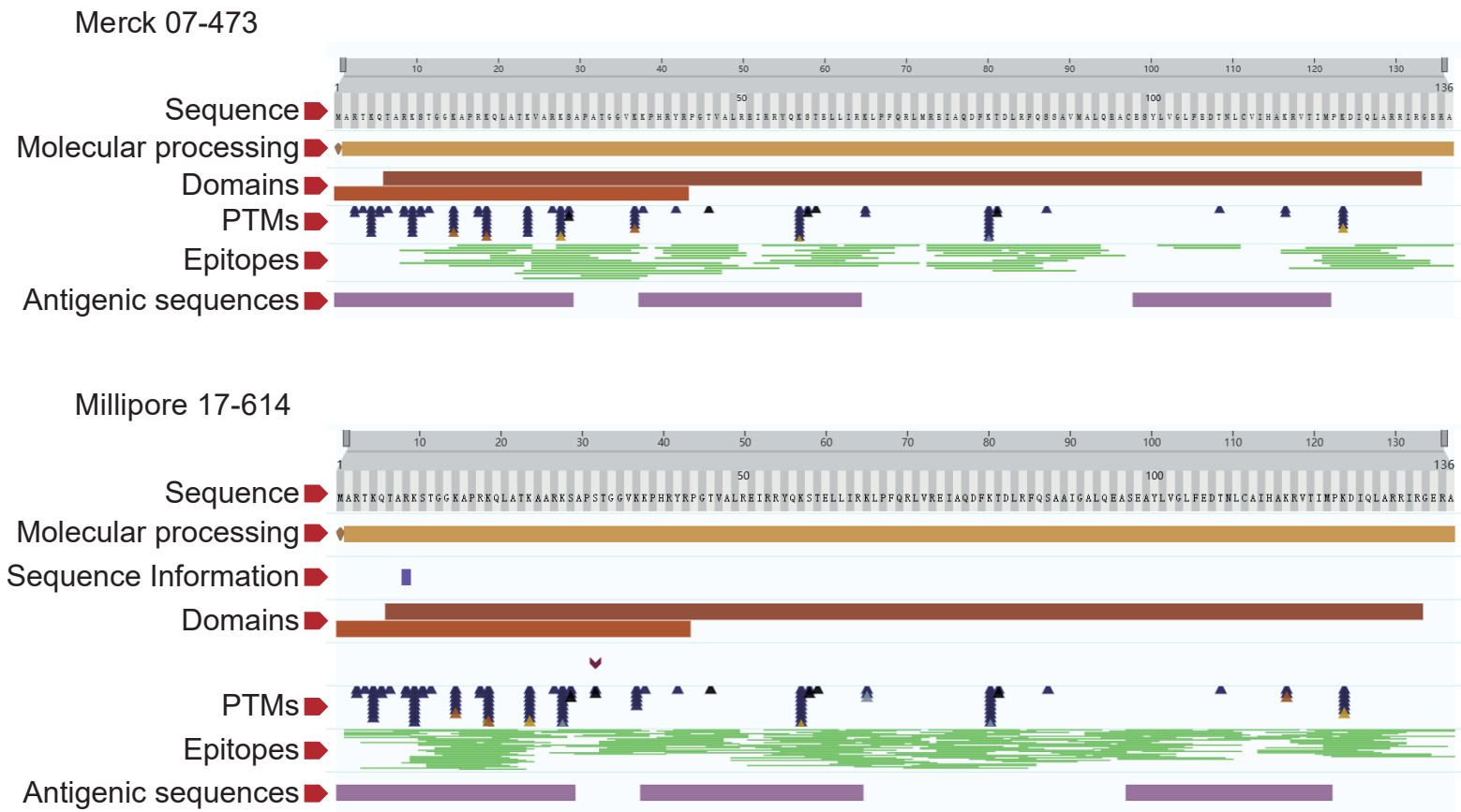

B

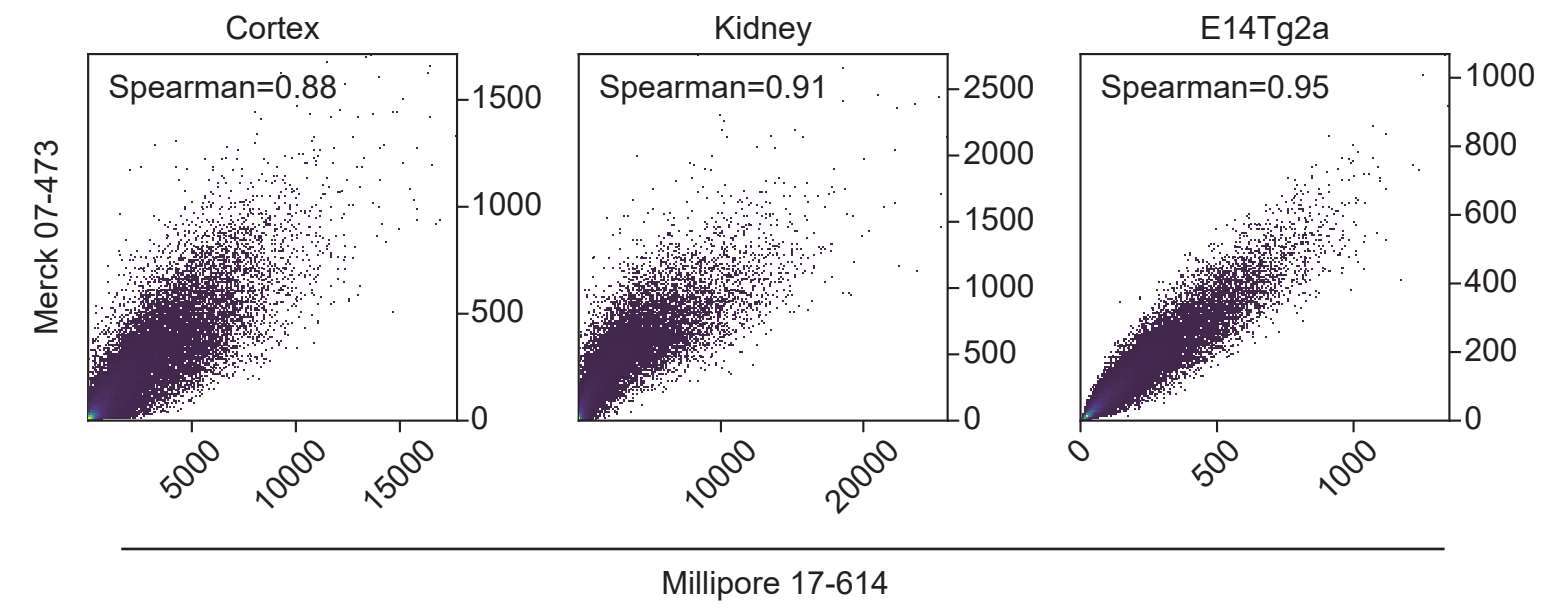

Supplement: Supplementary file 6 [file Image4.pdf]

A

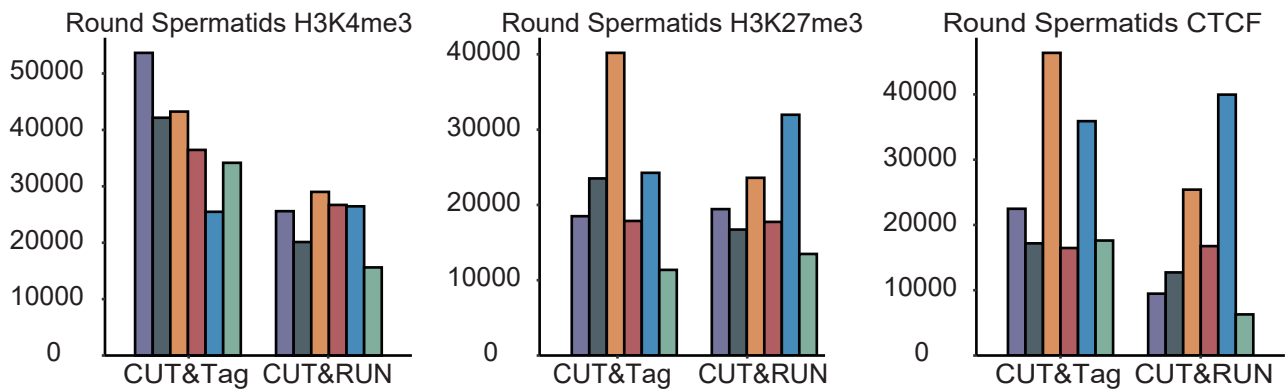

B

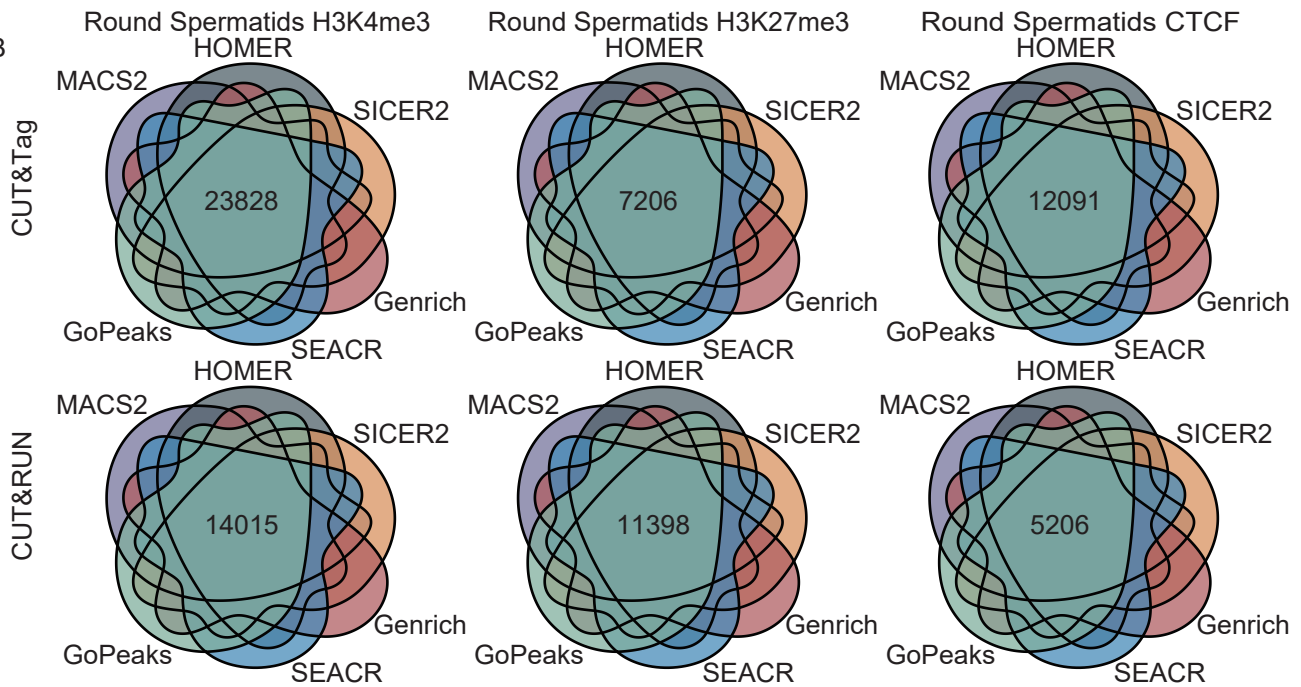

C

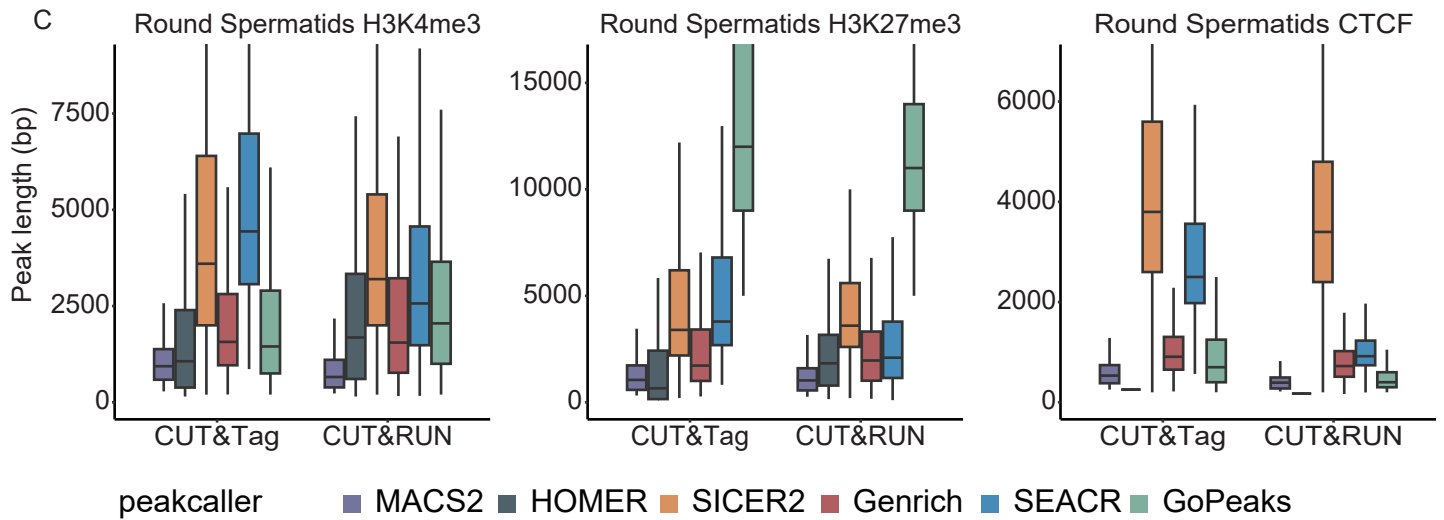

Supplement: Supplementary file 7 [file Image2.pdf]

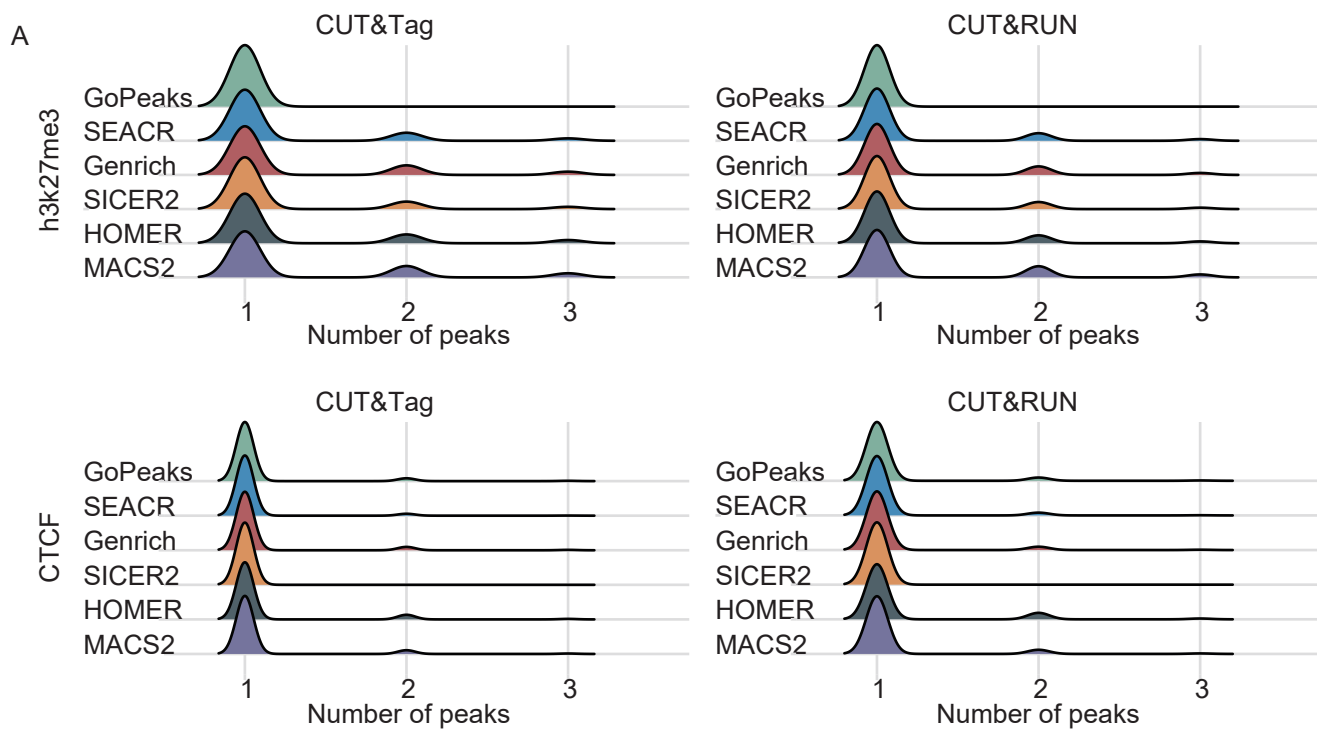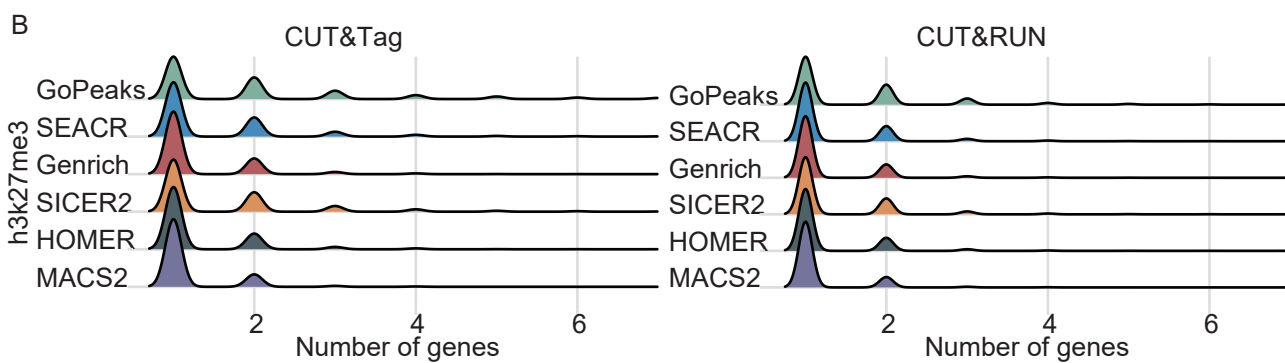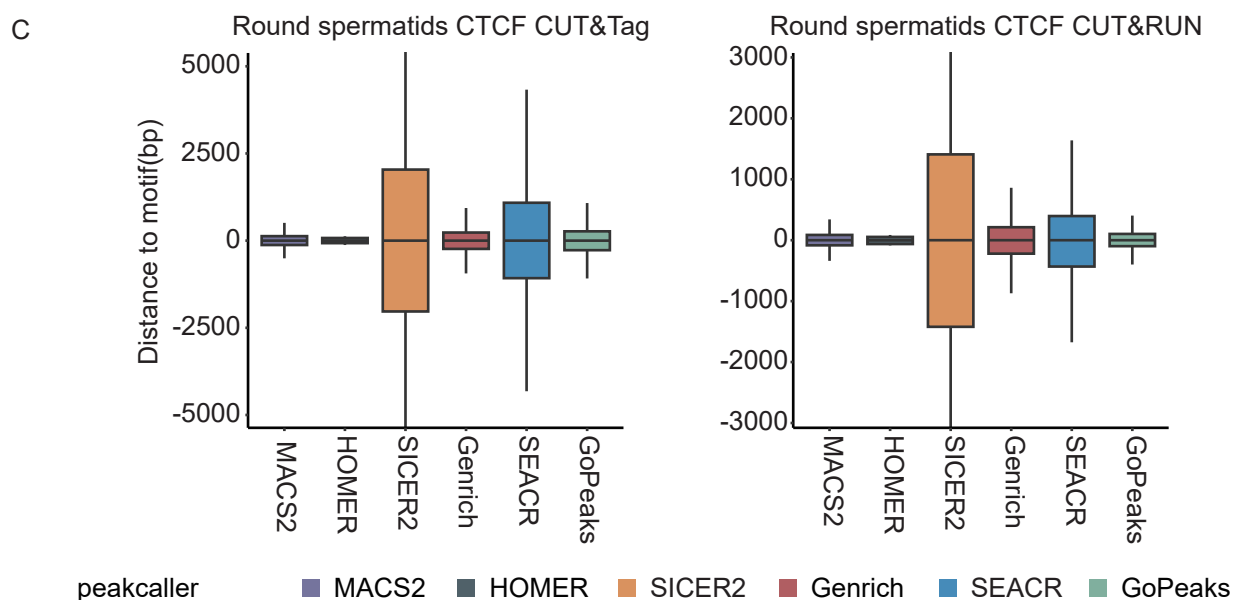

Supplement: Supplementary file 8 [file Image3.pdf]

A

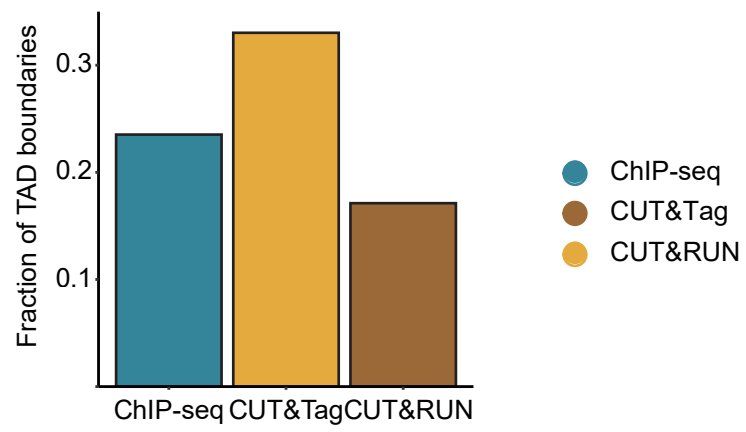

B

chr5: 124 MB - 138 MB

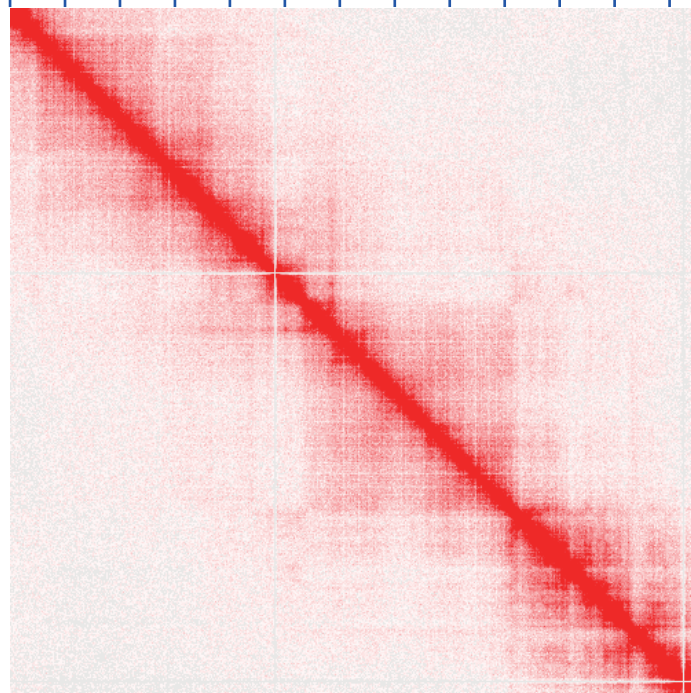

TAD boundary

chr5:134,452,827-134,544,334

TAD boundary

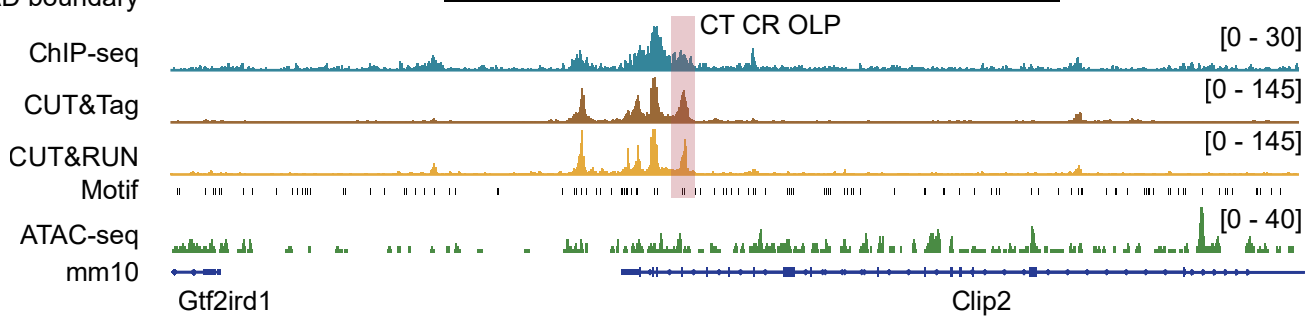

Supplement: Supplementary file 9 [file Image7.pdf]

H3K4me3

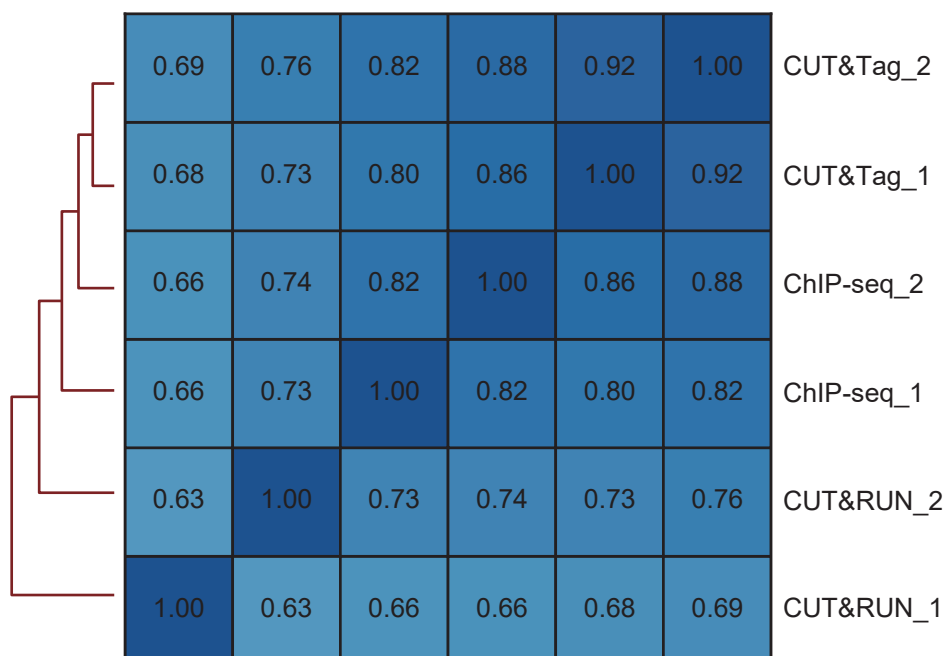

H3K27me3

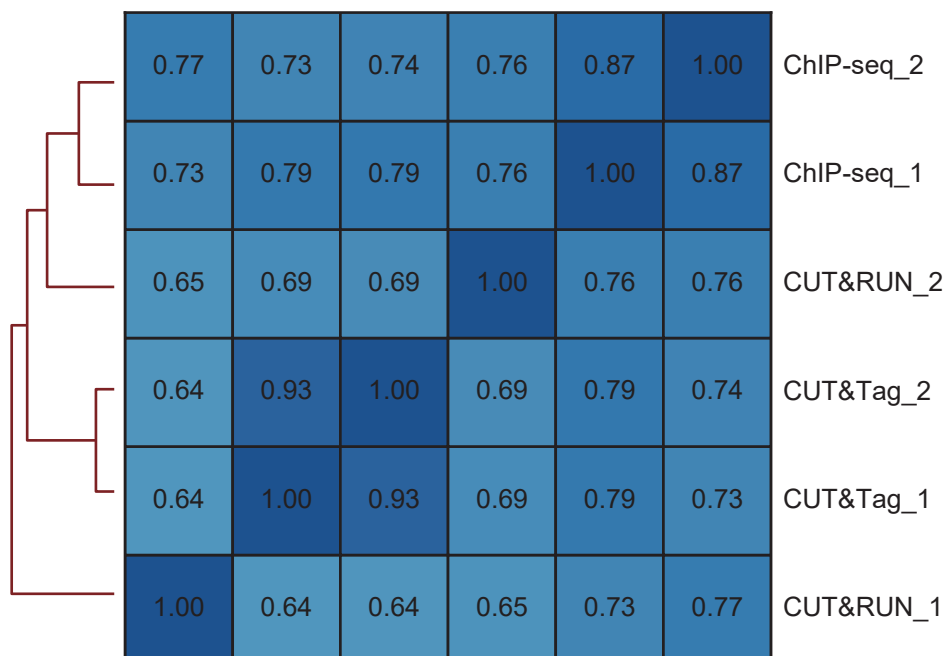

CTCF

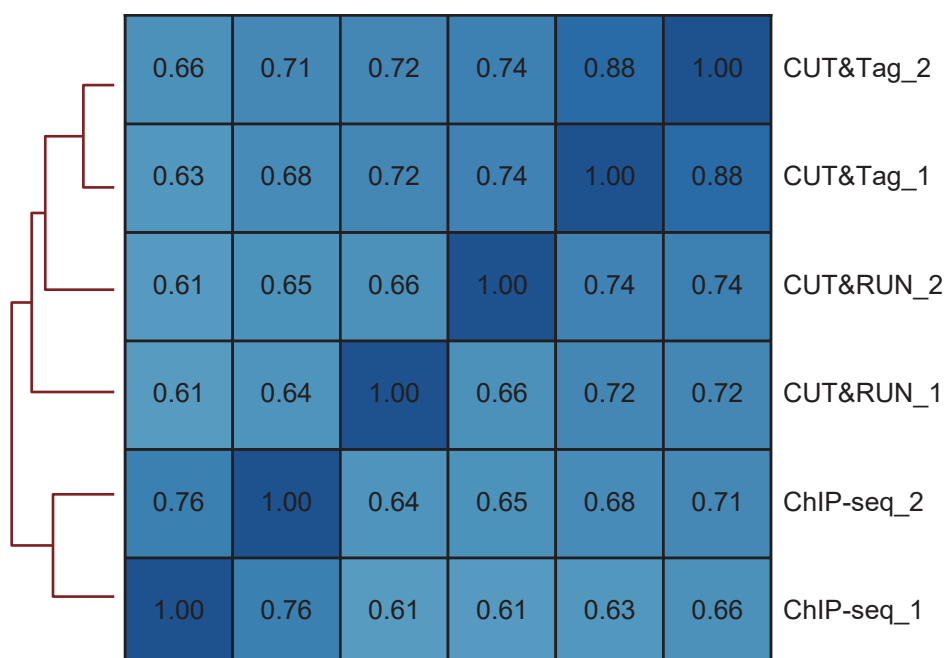

Supplement: Supplementary file 10 [file Image1.pdf]
