# Supplementary material for: Benchmark of chromatin–protein interaction methods in haploid round spermatids
Source: Front Cell Dev Biol. 2025 May 13;13:1572405. doi: 10.3389/fcell.2025.1572405 (PMC12106302; doi:10.3389/fcell.2025.1572405)
Supplement: Supplementary file 2 [file Table1.docx]

Table1 Number of MACS2 called peak

| Cell | Factor | Method | p=0.01 | Q=0.1 | Q=0.05 | Q=0.01 | Q=1e-5 |
| --- | --- | --- | --- | --- | --- | --- | --- |
| Round Spermatid | H3K4me3 | ChIP-seq | 80677 | 74890 | 70553 | 66411 | 51048 |
| Round Spermatid | H3K4me3 | CUT&Tag | 60976 | 52983 | 48970 | 44653 | 32507 |
| Round Spermatid | H3K4me3 | CUT&RUN | 57925 | 23435 | 21704 | 17196 | 8126 |
| Round Spermatid | H3K27me3 | ChIP-seq | 268160 | 123933 | 102716 | 69923 | 32459 |
| Round Spermatid | H3K27me3 | CUT&Tag | 154113 | 21126 | 17423 | 13733 | 8422 |
| Round Spermatid | H3K27me3 | CUT&RUN | 109950 | 20645 | 20153 | 17767 | 10258 |
| Round Spermatid | CTCF | ChIP-seq | 42627 | 12946 | 11344 | 9479 | 5420 |
| Round Spermatid | CTCF | CUT&Tag | 106036 | 27249 | 22481 | 15437 | 7715 |
| Round Spermatid | CTCF | CUT&RUN | 85067 | 11092 | 9477 | 6701 | 2170 |
| HEK293T | H3K4me3 | ChIP-seq | 17786 | 15671 | 15635 | 15275 | 14491 |
| HEK293T | H3K4me3 | CUT&Tag | 18610 | 16916 | 16873 | 16519 | 15524 |
| HEK293T | H3K4me3 | CUT&RUN | 23662 | 15860 | 15451 | 14283 | 12604 |
| E14Tg2a | H3K27me3 | ChIP-seq | 262906 | 113210 | 79323 | 33033 | 3673 |
| E14Tg2a | H3K27me3 | CUT&Tag | 167333 | 49913 | 43388 | 28301 | 12693 |
| E14Tg2a | H3K27me3 | CUT&RUN | 303816 | 44204 | 29025 | 16511 | 5039 |
| K562 | CTCF | ChIP-seq | 48752 | 31600 | 30589 | 28036 | 21598 |
| K562 | CTCF | CUT&Tag | 87112 | 35963 | 27777 | 22462 | 14278 |
| K562 | CTCF | CUT&RUN | 139134 | 61009 | 49296 | 43488 | 25860 |

Table2 Number of comparative peaks

| cell | factor | commonpeak | ChIP_CT_OLP | ChIP_CR_OLP | CT_CR_OLP | ChIP_unique | CT_unique | CR_unique |
| --- | --- | --- | --- | --- | --- | --- | --- | --- |
| Round Spermatid | H3K4me3 | 16609 | 10789 | 49 | 4 | 13097 | 600 | 18 |
| Round Spermatid | H3K27me3 | 11753 | 79 | 621 | 10 | 30411 | 385 | 17 |
| Round Spermatid | CTCF | 4130 | 171 | 84 | 766 | 477 | 2796 | 62 |
| HEK293T | H3K4me3 | 13421 | 698 | 178 | 939 | 4113 | 279 | 99 |
| E14Tg2a | H3K27me3 | 6875 | 119 | 137 | 71 | 755 | 2730 | 159 |
| K562 | CTCF | 12067 | 23 | 6371 | 5853 | 578 | 972 | 4939 |

Table3 Summary of Antibody in this research

| Accession | Cell type | Antibody | Method | Manufacturer | Catalog |
| --- | --- | --- | --- | --- | --- |
| \ | Round spermatid | H3K4me3 | CUT&Tag/CUT&RUN | Merck | 07-473 |
| \ | Round spermatid | H3K27me3 | CUT&Tag/CUT&RUN | Cell Signaling Technology | 9733s |
| \ | Round spermatid | CTCF | CUT&Tag/CUT&RUN | Abcam | ab70303 |
| GSE42629 | Round spermatid | H3K4me3 | ChIP-seq | Millipore | 17-614 |
| GSE42629 | Round spermatid | H3K27me3 | ChIP-seq | Millipore | 07-449 |
| GSE147536 | Round spermatid | CTCF | ChIP-seq | Santa Cruz Biotechnology | sc-271514 |
| GSE213209^[47]^ | HEK293T | H3K4me3 | ChIP-seq | Abclonal | A2357 |
| GSE223370^[48]^ | HEK293T | H3K4me3 | CUT&Tag | Abcam | ab8580 |
| GSE183730^[49]^ | HEK293T | H3K4me3 | CUT&RUN | Diagenode | C15410003 |
| GSE206735 | E14Tg2a | H3K27me3 | ChIP-seq | Cell Signaling Technology | 9733 |
| GSE253062 | E14Tg2a | H3K27me3 | CUT&Tag |  |  |
| GSE193910^[50]^ | E14Tg2a | H3K27me3 | CUT&RUN |  |  |
| GSE92881^[52]^ | K562 | CTCF | ChIP-seq | Millipore | 7729 |
| GSE124557^[29]^ | K562 | CTCF | CUT&Tag | Millipore | 7729 |
| GSE151326^[53]^ | K562 | CTCF | CUT&RUN | Millipore | 7729 |
